# Data on the impact of the blood sample collection methods on blood protein profiling studies

**DOI:** 10.1016/j.dib.2017.07.025

**Published:** 2017-07-14

**Authors:** Maria Ilies, Cristina Adela Iuga, Felicia Loghin, Vishnu Mukund Dhople, Thomas Thiele, Uwe Völker, Elke Hammer

**Affiliations:** a Department of Pharmaceutical Analysis, Faculty of Pharmacy, Iuliu Hațieganu University of Medicine and Pharmacy, no. 6 Louis Pasteur st., 400349 Cluj-Napoca, Romania; b Department of Functional Genomics, Interfaculty Institute of Genetics and Functional Genomics, University Medicine Greifswald, F.-L.-Jahn-Str. 15a, 17475 Greifswald, Germany; c Department of Proteomics and Metabolomics, MedFuture Research Center for Advanced Medicine, Iuliu Hațieganu University of Medicine and Pharmacy, no. 4-6 Louis Pasteur st., 400349 Cluj-Napoca, Romania; d Department of Toxicology, Faculty of Pharmacy Iuliu Hațieganu University of Medicine and Pharmacy, no. 6 Louis Pasteur st., 400349 Cluj-Napoca, Romania; e Institute of Immunology and Transfusion Medicine, University Medicine Greifswald, Sauerbruchstr., 17475 Greifswald, Germany; f DZHK (German Centre for Cardiovascular Research), partner site Greifswald, Greifswald, Germany

**Keywords:** LC-MS^E^ method data, Proteomics, Plasma, Serum, Proteomics

## Abstract

Complete blood protein profiles of 4 different blood sample collection methods (EDTA-, heparin- and citrate plasma and serum) were investigated and the data presented herein is an extension of the research article in Ilies et al. [1]. Specimens were depleted of 6 highly abundant proteins and protein profiling was assessed by nano-LC UDMS^E^. Exhaustive protein sets and protein abundances before and after depletion are presented in tables and figures. Also, the core protein set and the unique proteins for each sample collection method previously described [1] are disclosed.

**Specifications Table**

**Table:** 

Subject area	*Proteomics*
More specific subject area	*Clinical chemistry, Biomarker analysis, Blood proteome profiling*
Type of data	*Tables, figures (PDF file format)*
How data was acquired	*nano liquid chromatography (AQUITY UPLC M-CLASS, Waters Corporation) tandem mass spectrometry (Synapt G2Si mass spectrometer, WATERS Corporation)*
	*UDMS*^*E*^*data acquisition*
Data format	*Analyzed and processed data*
Experimental factors	*24 blood samples were drawn from 6 healthy young volunteers in serum tubes and plasma tubes containing EDTA, heparin, and citrate.*
Experimental features	*Serum and plasma was obtained after tube manufacturer's instructions and aliquots were stored at -80 °C until analysis. Protein profiles were analyzed before and after samples depletion of 6 high abundant proteins using a commercial MARS6 (Agilent Technologies) immunoaffinity based column. Prior to the mass spectrometric analysis, proteins were digested by trypsin and peptides were further analyzed and protein profiles investigated with respect to the sample collection method influence.*
Data source location	*Greifswald, Germany*
Data accessibility	*Data is with article*

**Value of the data**

• Data shows a comprehensive evaluation of the different blood sample collection methods on 6 high abundant proteins and their depletion efficiency using immunoaffinity MARS6 column which can be used for future investigations on blood high abundant proteins and depleted fractions.
• Individual protein abundances, their presence and variance in the samples collected with different methods after depletion are of potential value to determine which sampling method to be used for proteomics investigations.
• Data presents an all-inclusive set of information on the methods applied to evaluate the impact of different blood sample collection methods on protein profiling studies and can be used as benchmark for future blood protein profiling studies.

## 1 Data

**Fig. 1:**
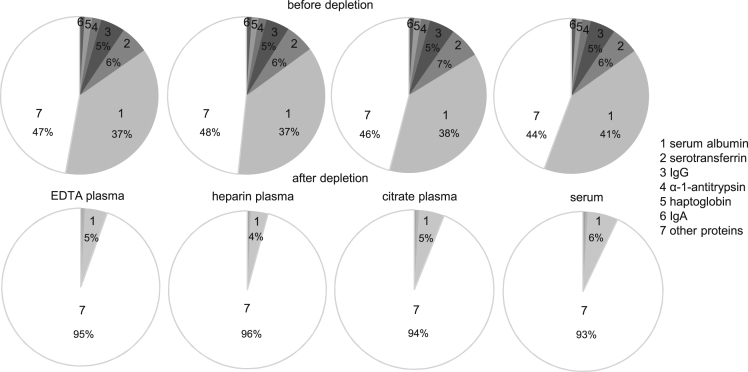
HAP abundance before and after depletion.

**Fig. 2:**
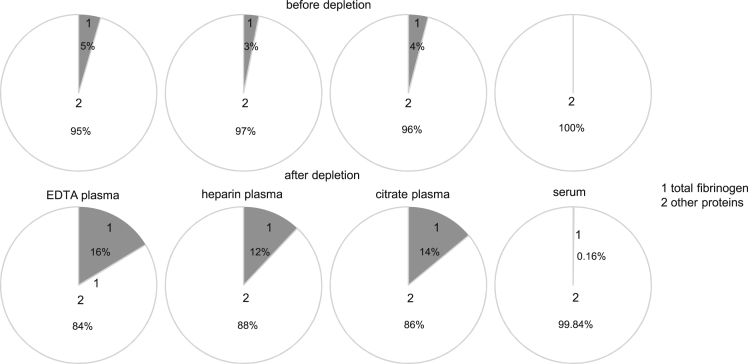
Total fibrinogen abundance before and after depletion of HAP.

**Fig. 3:**
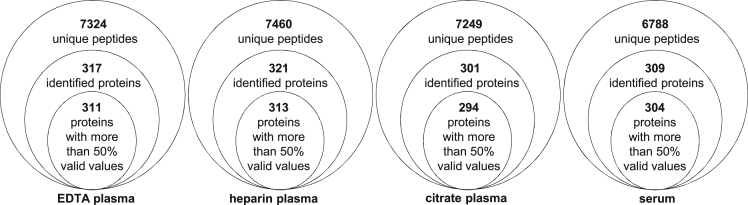
Global overview on the identified peptides and quantified proteins.

In this Data in Brief article we provide detailed information on blood protein profiling as an extension of the results reported in Ref. [1], 24 blood specimens were collected from 6 healthy and young volunteers in different sample collection tubes for serum and plasma. Tubes characteristics and the subsequent sample preparation are presented in Table 1. For the blood protein profiling a nanoLC-UDMS^E^ method and standard search parameters were employed. Detailed description of methods can be found in Ref. [1] and its supplementary methods. 6 highly abundant blood proteins, namely serum albumin, immunoglobulin gamma, immunoglobulin alpha, serotransferrin, haptoglobin, and alpha-1-antitrypsin, were depleted by using a commercially available immunoaffinity depletion column. A detailed overview on depletion efficiency based on protein abundances for all sample collection methods is presented in Table 2. The distribution of the high abundant proteins before and after depletion is presented in Fig. 1 and more specific, fibrinogen coverage is shown in Fig. 2. Data regarding number of identified peptides and relatively quantified proteins for all sample types after depletion is shown in Fig. 3. Also, a top 10 list of the most abundant unique proteins for each of the EDTA-, heparin-, citrate plasma and serum samples is given in Table 3. The complete list of all relatively quantified proteins over all samples including their occurrence in the protein core set or as unique proteins interpreted in detail previously [1], can be found in the Supplementary material with data on individual sample abundance, mean abundance for each sample collection method and the abundance based coefficient of variation after depletion.

**Table 1:** Blood sample collection tubes characteristics.

**Blood product**	**Serum**	**Plasma**		
**Tube type**	Plastic SST™ II Advance	Plastic K2EDTA	Glass Citrate	Glass sodium heparin
**Cat. No./NHS code**	367954/KFK114	367873/KFK286	367691/KFK186	367876/KFK279
**Additive (concentration)**	Silica (clot activator)/gel	Potassium EDTA	Buffered sodium citrate (0,105 M)	Sodium heparin (17 IU/mL blood)
**Volume (mL)**	5	6	4.5	6
**Mixing recommendation**	Gently inverted 180° and back	Gently inverted 180° and back	Gently inverted 180° and back	Gently inverted 180° and back
	5–6 times	8–10 times	3–4 times	8–10 times

**Table 2:** Summary of depletion efficiency.

Mean protein abundance	**EDTA plasma**		**Heparin plasma**		**Citrate plasma**		**Serum**	
	Before depletion	After depletion	Before depletion	After depletion	Before depletion	After depletion	Before depletion	After depletion
All proteins	40568018.74	44741390.04	35119594.07	52949084.17	40671622.40	69872,370.90	54995897.89	45191163.47
α-1-antitrypsin	757196.34	14259.97	584561.17	23153.90	764890.57	22854.17	1006489.65	29692.67
Haptoglobin	631767.39	18029.34	487822.58	41219.67	596917.79	56161.72	689948.19	41259.85
Ig A	429327.89	44422.15	380247.33	23957.12	431941.12	51775.29	550978.46	18885.19
Ig G 1–4	2013660.86	91452.63	1677118.63	101491.82	2145431.99	168092.78	2856603.51	124679.28
Serotransferrin	2369837.17	213742.01	2096978.58	183333.16	2651548.54	364344.32	3224215.08	202618.47
Serum albumin	15235797.33	2056072.75	12925303.83	1866019.81	15394810.67	3600066.33	22315530.67	2807855.77
Other proteins	19130431.76	42303411.19	16967561.94	50709908.69	18686081.72	65609076.29	24352132.33	41966172.25
Total fibrinogen	1839721.65	7325737.50	1108301.52	6370617.46	1657777.20	9817167.88	0.00	71699.88

**Table 3:** TOP 10 abundant proteins exclusively identified per sampling method.

**Accession**	**Entry name**	**TOP10_EDTA plasma Protein names**	**Secreted/ leakage**	**EDTA 1**	**EDTA2**	**EDTA3**	**EDTA4**	**EDTA5**	**EDTA6**	**Mean**	**CV**
O75410	TACC1	Transforming acidic coiled-coil-containing protein 1	Leakage	49174	65077	109984	54229	65833	39035	63889	0.39
Q9P2D6	F135A	Protein FAM135A	Not specified	47425	51140	9049	33084	57915	46010	40770	0.43
Q99683	M3K5	Mitogen-activated protein kinase kinase kinase 5	leakage	37558	44893	34790	36856	52333	37776	40701	0.16
Q9P0W8	SPAT7	Spermatogenesis-associated protein 7	Leakage	21640	32866	28811	17938	32732	19729	25619	0.26
Q9BYW2	SETD2	Histone-lysine N-methyltransferase SETD2	Leakage	22027	26583	21030	22561	30931	20783	23986	0.17
Q15573	TAF1A	TATA box-binding protein-associated factor RNA polymerase I subunit A	Leakage	14671	20078	22199	21065	27359	18243	20603	0.21
P15813	CD1D	Antigen-presenting glycoprotein CD1d	Leakage	13395	17280	13398	14071	20923	15275	15724	0.19
O14950	ML12B	Myosin regulatory light chain 12B	Not specified	10508	9974	11784	13291	8753	9291	10600	0.16
Q5TBE3	CI153	Uncharacterized protein C9orf153	Not specified	6183	7639	8439	7543	11014	7128	7991	0.21
Q8N4P6	LRC71	Leucine-rich repeat-containing protein 71	Not specified	10463	8450	5703	7123	6758	6869	7561	0.22
**Accession**	**Entry name**	**TOP10_heparin plasma Protein names**	**Secreted/ leakage**	**Heparin1**	**Heparin2**	**Heparin3**	**Heparin4**	**Heparin5**	**Heparin6**	**Mean**	**CV**

Q8WUY3	PRUN2	Protein prune homolog 2	Leakage	160723	206288	192573	306294	247158	283487	232754	0.24
Q15811	ITSN1	Intersectin-1	Leakage	50959	63100	53081	48220	90431	34339	56688	0.33
Q00610	CLH1	Clathrin heavy chain 1	Leakage	15586	14728	18562	20815	22935	20262	18815	0.17
P24043	LAMA2	Laminin subunit alpha-2	Secreted	11392	12288	14534	17688	16610	15376	14648	0.17
Q96RE9	ZN300	Zinc finger protein 300	Leakage	7134	11365	11937	20051	15033	15472	13499	0.33
Q9P0W5	SCHI1	Schwannomin-interacting protein 1	Leakage	17022	10273	10231	14051	10710	10656	12157	0.23
Q5RL73	RBM48	RNA-binding protein 48	Not specified	5454	8424	11531	14460	8906	21118	11649	0.48
Q9P219	DAPLE	Protein Daple	Leakage	7162	10200	14709	10857	11183	10820	10822	0.22
Q8N3R3	TCAIM	T-cell activation inhibitor, mitochondrial	Leakage	13138	11101	8965	8622	9601	8057	9914	0.19
Q7LG56	RIR2B	Ribonucleoside-diphosphate reductase subunit M2 B	Leakage	5452	8109	8708	10250	8552	9235	8384	0.19

**Accession**	**Entry name**	**TOP10_citrate plasma Protein names**	**Secreted/ leakage**	**Citrate1**	**Citrate2**	**Citrate3**	**Citrate4**	**Citrate5**	**Citrate6**	**Mean**	**CV**
Q9P2M7	CING	Cingulin	Leakage	25422	20003	21886	23546	30961	24534	24392	0.15
P12259	FA5	Coagulation factor V	Secreted	23814	17933	19660	25752	18389	25697	21874	0.17
Q9P2F6	RHG20	Rho GTPase-activating protein 20	Not specified	23589	22007	16027	12855	27896	20017	20398	0.26
Q8N4C7	STX19	Syntaxin-19	Leakage	28854	20451	15902	13099	22999	13806	19185	0.32
P01036	CYTS	Cystatin-S	secreted	11101	15659	13394	13586	12003	13467	13201	0.12
Q9UHR6	ZNHI2	Zinc finger HIT domain-containing protein 2	Not specified	19443	16822	4967	3913	10581	11941	11278	0.55
Q96RG2	PASK	PAS domain-containing serine/threonine-protein kinase	Leakage	6479	5955	8749	6906	9560	7207	7476	0.19
P82970	HMGN5	High mobility group nucleosome-binding domain-containing protein 5	Leakage	9548	8133	7295	6810	7588	5103	7413	0.20
Q9Y275	TN13B	Tumor necrosis factor ligand superfamily member 13B	Secreted	7560	5901	9552	8230	5887	5013	7024	0.24
Q8TBF8	FA81A	Protein FAM81A	Not specified	2397	14042	4879	6685	5055	6287	6558	0.60
**Accession**	**Entry name**	**TOP10_serum Protein names**	**Secreted/ leakage**	**Serum 1**	**Serum2**	**Serum3**	**Serum4**	**Serum5**	**Serum6**	**Mean**	**CV**

P04275	VWF	von Willebrand factor	Secreted	171296	202739	192598	166870	183833	187044	184063	0.07
O95602	RPA1	DNA-directed RNA polymerase I subunit RPA1	Leakage	118817	176926	184007	192232	145288	177692	165827	0.17
Q9ULI0	ATD2B	ATPase family AAA domain-containing protein 2B	Leakage	51935	64557	22306	53282	62255	57282	51936	0.30
Q96HQ0	ZN419	Zinc finger protein 419	Leakage	36426	50320	48458	36400	54318	39176	44183	0.18
P07996	TSP1	Thrombospondin-1	Leakage	44367	39433	21407	42369	47867	37343	38797	0.24
Q9BS31	ZN649	Zinc finger protein 649	Leakage	23893	41507	27030	51443	37056	43081	37335	0.28
A6NET4	OR5K3	Olfactory receptor 5K3	Leakage	38230	37448	43819	30573	25303	33517	34815	0.19
Q8WXX0	DYH7	Dynein heavy chain 7, axonemal	Leakage	19153	26320	19664	22016	33037	19816	23334	0.23
Q7Z443	PK1L3	Polycystic kidney disease protein 1-like 3	Leakage	12597	7542	19654	27879	14351	4420	14407	0.59
P98196	AT11A	Probable phospholipid-transporting ATPase IH	Leakage	15647	10307	12192	15634	14152	17414	14224	0.18

## 2 Experimental design, materials and methods

Experimental design and the materials and methods have been reported previously [1].

## Appendix A Supplementary material

Overview on all sample collection methods relatively quantified proteins after depletion.
