# Supplementary material for: Data on the impact of the blood sample collection methods on blood protein profiling studies
Source: Data Brief. 2017 Jul 14;14:313–9. doi: 10.1016/j.dib.2017.07.025 (PMC5544472; doi:10.1016/j.dib.2017.07.025)
Supplement: Supplementary file 1 — Supplementary material [file mmc2.docx]

**Conflict of interest**

The authors declare that they have no conflict of interest.
