# Supplementary material for: Data on the impact of the blood sample collection methods on blood protein profiling studies
Source: Data Brief. 2017 Jul 14;14:313–9. doi: 10.1016/j.dib.2017.07.025 (PMC5544472; doi:10.1016/j.dib.2017.07.025)
Supplement: Supplementary file 2 — Overview on all sample collection methods relatively quantified proteins after depletion. [file mmc1.pdf]

Supplementary material Overview on all sample collection methods relatively quantified proteins after depletion

Nr	Accession	Entry name	Protein names	secreted/ leakage	core set	unique EDTA	unique heparin	unique citrate	unique serum	EDTA plasma samples EDTA_1	EDTA_2	EDTA_3	EDTA_4	EDTA_5	EDTA_6	Mean abundance	CV	heparin plasma samples heparin_1	heparin_2	heparin_3	heparin_4	heparin_5	heparin_6	Mean abundance	CV	citrate plasma samples citrate_1	citrate_2	citrate_3	citrate_4	citrate_5	citrate_6	Mean abundance	CV	serum samples serum_1	serum_2	serum_3	serum_4	serum_5	serum_6	Mean abundance	CV
1	P01023	A2MG_HUMAN	Alpha-2-macroglobulin	secreted	x					4316761.50	5766907.00	7003013.00	8030537.50	8156051.50	5502663.00	6462655.58	0.24	5492022.00	6479655.50	5288168.00	8500964.00	6812182.50	6451503.00	6411912.17	0.18	7997181.00	8410630.00	6986378.50	10024459.00	8361140.50	8597543.00	8396222.00	0.12	5348551.50	8001203.50	6947245.00	9327345.00	8011260.00	9249502.00	7814184.50	0.19
2	P01024	CO3_HUMAN	Complement C3	secreted	x					4217113.00	4794088.00	5289195.50	4627705.50	5437685.00	3634529.25	4666719.38	0.14	6620916.00	5842467.50	5714171.00	6266736.00	5936969.50	5615602.00	5999477.00	0.06	9456614.00	8983310.00	7827573.50	7783685.50	7984749.00	7608675.00	8274101.17	0.09	5153105.50	6438793.50	6082043.50	5744775.50	5801346.50	5977817.50	5866313.67	0.07
3	P02647	APOA1_HUMAN	Apolipoprotein A-I	secreted	x					3285028.25	3681459.25	5054543.50	3245844.75	5676832.00	2435117.50	3896470.88	0.31	2884890.00	2754011.25	2871730.75	2374609.75	3532361.75	2182148.75	2766625.38	0.17	5468897.50	4533579.00	4291045.50	3721592.25	5127792.00	3402457.00	4424227.21	0.18	2039911.50	2599474.25	2420339.75	2228058.75	2908565.50	2256624.75	2408829.08	0.13
4	P02675	FIBB_HUMAN	Fibrinogen beta chain	secreted	x					2712859.75	2995402.75	2824416.25	2779879.75	3665294.25	2841502.00	2969892.46	0.12	2881945.75	3117478.75	2566007.25	2663938.75	2811251.75	2654895.75	2782586.33	0.07	4527366.50	4214724.50	3478601.75	4155317.25	4822157.00	4677126.50	4312548.92	0.11	10841.38	13298.57	9734.24	6551.17	7306.78	7810.21	9257.06	0.27
5	P02679	FIBG_HUMAN	Fibrinogen gamma chain	secreted	x					2266602.00	2545914.00	2502777.50	2443039.00	3113360.25	2324097.50	2532631.71	0.12	2186665.00	2442741.25	1869724.50	2076868.63	2150866.50	2029224.63	2126015.08	0.09	3706382.00	3266745.75	2612731.00	3117708.00	3421258.50	3447420.00	3262040.88	0.11	12873.49	11948.03	10843.04	8339.87	12795.72	9481.99	11047.02	0.17
6	P02768	ALBU_HUMAN	Serum albumin	secreted	x					1533686.75	2081836.63	3365307.50	1767179.25	2311734.00	1276692.38	2056072.75	0.36	1399239.75	1525328.63	2006343.88	2009838.38	2257395.50	1997972.75	1866019.81	0.18	4256458.00	4005760.50	3887050.00	3565486.25	2870161.00	3015482.25	3600066.33	0.15	2619699.75	2715270.50	3057620.00	2226872.50	4249299.50	1978372.38	2807855.77	0.29
7	P04114	APOB_HUMAN	Apolipoprotein B-100	secreted	x					1948543.25	1894844.50	1506656.63	2691365.25	1803263.38	2319679.50	2027392.08	0.21	3163830.00	2584346.00	2254482.75	3957654.25	2110084.25	3884255.50	2992442.13	0.27	3876033.25	3419223.00	2622704.25	4476150.00	2573814.75	4619907.50	3597972.13	0.25	2329754.00	2620381.50	2242213.75	3406767.00	1977536.25	3936583.50	2752206.00	0.28
8	P02671	FIBA_HUMAN	Fibrinogen alpha chain	secreted	x					1661901.88	1779607.63	1867988.63	1637405.13	2283713.50	1708663.25	1823213.33	0.13	1496068.75	1633467.25	1406886.88	1355902.25	1510282.13	1369489.00	1462016.04	0.07	2523416.75	2204247.25	1874754.75	2203712.25	2286641.50	2362696.00	2242578.08	0.10	52303.72	72424.13	49673.48	44157.87	40773.88	49041.71	51395.80	0.22
9	P02790	HEMO_HUMAN	Hemopexin	secreted	x					1458362.75	1741557.13	1669381.63	1696909.25	2135936.75	1283869.75	1664336.21	0.17	2036594.88	1958465.38	1672579.50	2009626.63	2160129.50	1871899.75	1951549.27	0.09	3354627.50	3055317.75	2114542.00	2524564.00	2785577.75	2563560.25	2733031.54	0.16	1473814.13	1869004.13	1402819.38	1483151.38	1761319.63	1796201.13	1631051.63	0.12
10	P00450	CERU_HUMAN	Ceruloplasmin	secreted	x					610515.25	1063926.75	889022.25	802599.81	999965.94	963584.00	888269.00	0.18	1217391.13	1766531.25	1388632.00	1452223.88	1472741.75	1915915.25	1535572.54	0.17	1394791.63	2069309.50	1529931.13	1696452.75	1681498.75	2237968.50	1768325.38	0.18	780148.19	1495375.75	1102526.25	1022111.13	1068458.25	1594856.50	1177246.01	0.26
11	P02774	VTDB_HUMAN	Vitamin D-binding protein	secreted	x					849735.25	811727.88	867884.19	723884.38	918615.56	654698.69	804424.32	0.12	1267655.13	1170174.63	1263604.38	1103816.13	1169781.50	1267074.88	1207017.77	0.06	1344328.00	1324615.63	1439648.75	1310298.13	1271621.13	1355536.00	1341007.94	0.04	1078823.50	1107237.88	1251993.00	1016866.94	1070574.38	1215309.13	1123467.47	0.08
12	P02652	APOA2_HUMAN	Apolipoprotein A-II	secreted	x					662333.75	644481.25	945051.06	635677.69	775377.19	531821.25	699123.70	0.20	828507.31	705944.63	742644.44	786117.19	703517.31	791320.88	759675.29	0.07	1429194.38	1221060.38	1216859.25	1163783.50	980111.13	1100175.38	1185197.33	0.13	710880.56	770642.94	876210.31	671643.63	687934.81	825913.38	757204.27	0.11
13	P02766	TTHY_HUMAN	Transthyretin	secreted	x					763436.50	685289.50	824346.06	645795.25	722010.31	478770.75	686608.06	0.17	1774275.50	1344307.25	1529973.13	1338038.63	1230540.75	1237490.00	1409104.21	0.15	1770531.38	1088636.50	1161522.75	1085993.38	1078039.63	1004525.00	1198208.10	0.24	934345.75	922059.31	878408.44	743881.63	698911.00	796106.94	828952.18	0.12
14	P08603	CFAH_HUMAN	Complement factor H	secreted	x					676780.25	689794.69	539103.69	623335.81	685061.56	583408.94	632914.16	0.10	1009480.50	969231.00	808583.44	932715.00	851836.06	891900.56	910624.43	0.08	1189120.38	1006191.81	851954.88	1083410.13	1152071.88	1066763.25	1058252.05	0.11	845596.38	1006770.88	804864.19	922087.69	853754.63	940197.13	895545.15	0.08
15	P02749	APOH_HUMAN	Beta-2-glycoprotein 1	secreted	x					644678.00	623521.31	592029.25	542162.44	588410.00	380005.72	561801.12	0.17	831297.38	701133.75	678937.13	702373.50	668300.31	478232.25	676712.39	0.17	1242502.88	970748.69	886259.25	855430.44	957860.50	657564.44	928394.36	0.21	731235.19	804364.19	666332.31	680000.19	668830.31	580073.50	688472.61	0.11
16	Q14624	ITIH4_HUMAN	Inter-alpha-trypsin inhibitor heavy chain H4	secreted	x					449598.53	469730.50	513039.41	475960.41	586938.19	386624.47	480315.25	0.14	717883.25	594706.88	722021.38	671399.56	713197.75	700106.81	686552.60	0.07	909045.75	760010.00	804994.13	771435.38	849949.81	776626.25	812010.22	0.07	554847.38	576010.44	693144.31	594303.50	587103.81	624176.13	604930.93	0.08
17	P01042	KNG1_HUMAN	Kininogen-1	secreted	x					453354.94	437421.50	438465.41	377023.00	509945.56	393197.44	434901.31	0.11	798963.38	643755.69	612478.38	589077.06	678540.63	661068.88	663980.67	0.11	1030598.88	819442.38	707192.81	700032.25	804632.69	801896.00	810632.50	0.15	593140.50	639418.50	541395.69	514502.66	604816.13	679199.56	595412.17	0.10
18	P04217	A1BG_HUMAN	Alpha-1B-glycoprotein	secreted	x					387279.16	474705.25	532227.25	405580.03	453521.22	313649.59	430827.08	0.16	459859.66	473025.75	505340.59	458260.38	460421.97	465346.13	470375.74	0.04	771993.75	807536.56	710459.94	672842.31	783907.25	688277.06	739169.48	0.08	384262.59	506447.63	457537.91	383675.75	383524.13	431310.41	424459.73	0.12
19	P19823	ITIH2_HUMAN	Inter-alpha-trypsin inhibitor heavy chain H2	secreted	x					470899.09	447378.69	451155.69	405483.41	410354.47	369050.06	425720.23	0.09	616677.56	539988.69	676551.44	566040.69	522048.66	651448.31	595459.22	0.10	809241.56	630350.38	758070.69	682551.69	656975.56	748480.19	709741.68	0.09	520873.78	541263.75	601832.56	539084.75	494618.25	612811.38	551747.41	0.08
20	P01008	ANT3_HUMAN	Antithrombin-III	secreted	x					463490.56	444770.66	431325.56	391659.69	431885.22	286384.31	408252.67	0.16	674764.31	621926.88	681852.06	557107.81	600403.00	502915.69	606494.96	0.11	863339.38	791899.75	781828.88	749822.56	734418.19	633418.94	759121.28	0.10	499565.22	531265.06	522786.28	461253.78	455160.94	416957.16	481164.74	0.09
21	P02763	A1AG1_HUMAN	Alpha-1-acid glycoprotein 1	secreted	x					279941.19	474010.47	401051.31	554422.94	422280.75	269444.91	400191.93	0.28	319491.56	539810.88	533170.06	554989.56	484935.75	450344.03	478790.31	0.18	432873.63	658534.81	583299.44	780872.31	608851.75	556249.69	603446.94	0.19	263884.28	413262.69	409553.72	446729.31	364956.88	396111.91	382416.45	0.17
22	P19827	ITIH1_HUMAN	Inter-alpha-trypsin inhibitor heavy chain H1	secreted	x					396812.75	385973.00	450705.16	350854.00	390240.59	340147.84	385788.89	0.10	674396.19	559146.00	583894.06	614812.94	509358.56	610442.19	592008.32	0.09	770406.94	648538.00	651953.50	587208.88	583028.56	687760.38	654816.04	0.11	528099.06	556924.38	575895.31	513956.13	446020.63	631290.25	542030.96	0.12
23	P02765	FETUA_HUMAN	Alpha-2-HS-glycoprotein	secreted	x					418258.88	380846.94	366637.69	268402.31	358482.66	381388.41	362336.15	0.14	882830.00	765041.69	734003.06	561030.81	786808.00	953776.06	780581.60	0.17	935116.50	788416.50	604210.63	525108.81	808436.13	845769.13	751179.53	0.21	594225.81	653924.94	570520.00	420126.59	620585.13	759492.81	603145.88	0.18
24	P06727	APOA4_HUMAN	Apolipoprotein A-IV	secreted	x					407849.44	312006.72	366536.66	351264.91	442981.59	233184.22	352313.92	0.21	624386.56	421060.81	445129.75	502869.28	528144.25	363162.94	480792.27	0.19	664805.06	377938.53	381651.06	468892.66	561364.19	359928.91	469096.73	0.26	463578.09	375163.56	340657.25	436231.59	437079.69	337821.28	398421.91	0.14
25	P05751	CFAB_HUMAN	Complement factor B	secreted	x					330063.53	339221.03	367278.31	314976.50	409801.34	281207.00	340424.62	0.13	507258.44	472792.53	515178.19	430023.94	470868.59	470906.34	478338.01	0.06	610573.56	497526.75	481069.75	463243.31	604383.31	465548.50	520390.86	0.13	391055.09	471034.56	481084.13	415380.28	461845.19	461866.59	446741.31	0.08
26	P00734	THRB_HUMAN	Prothrombin	secreted	x					361888.41	371644.31	271149.28	320579.44	353287.00	279583.94	326355.40	0.13	535532.69	518175.72	447384.09	472722.56	454977.56	438278.66	477845.21	0.08	823483.06	608424.06	514005.81	640139.19	698652.44	603031.13	647955.95	0.16	401353.97	464467.13	352977.97	408585.56	400000.31	426895.91	408763.47	0.09
27	P04004	VTNC_HUMAN	Vitronectin	secreted	x					322625.34	319662.09	326123.03	253928.11	359181.19	297048.22	313094.66	0.11	363590.63	345008.19	359501.97	262379.59	352225.75	386733.56	344906.61	0.12	548099.19	431788.03	423097.56	366703.31	469192.91	479015.25	452982.71	0.14	333601.63	373892.91	333636.44	289064.47	362266.09	397631.38	348348.82	0.11
28	P00747	PLMN_HUMAN	Plasminogen	secreted	x					321613.13	323440.38	214779.88	262866.91	329888.94	309948.22	293756.24	0.16	467579.13	477725.81	420686.50	385130.16	434432.47	442119.09	437945.53	0.08	482721.09	438040.06	436009.88	474517.13	564196.69	515719.22	485200.68	0.10	400213.38	428457.22	400763.44	385910.69	439105.22	505559.25	426668.20	0.10
29	P02748	CO9_HUMAN	Complement component C9	secreted	x					234846.09	296957.56	327707.75	255445.69	419718.09	200843.33	289253.09	0.22	89567.97	134379.52	112067.82	119760.68	137803.52	125327.46	123924.87	0.15	73441.98	127960.75	112841.52	122226.52	148193.55	111643.50	115927.21	0.22	274572.25	391726.25	318764.84	319947.91	409780.88	310059.53	338408.86	0.16
30	P01011	AACT_HUMAN	Alpha-1-antichymotrypsin	secreted	x					221202.72	262836.03	352254.38	279785.09	271284.47	182411.27	261628.99	0.22	334375.00	336198.00	398369.00	387938.41	403277.66	278592.97	356458.51	0.14	536650.44	533932.25	556453.13	516164.47	571378.81	381967.56	516091.11	0.13	307238.66	430911.38	450683.88	436304.38	370871.03	314879.69	385148.17	0.17
31	P05155	IC1_HUMAN	Plasma protease C1 inhibitor	secreted	x					241394.64	226124.55	294390.03	285037.00	284320.91	197179.08	254741.03	0.15	465640.53	344572.09	416813.44	483277.38	415253.06	358210.41	413961.15	0.13	563625.19	421920.66	446322.88	508680.09	413139.75	419358.81	462174.56	0.13	284638.25	279320.06	317666.38	331156.19	300919.47	303701.66	302900.33	0.06
32	P02787	TRFE_HUMAN	Serotransferrin	secreted	x					185067.00	238395.41	200736.19	215080.75	277259.16	165023.58	213742.01	0.19	129611.13	156072.36	182975.13	187277.97	263689.88	180372.48	183333.16	0.25	350110.16	301556.16	340955.09	414507.81	465632.34	313304.34	364344.32	0.17	145663.92	163729.02	188049.00	156622.19	433481.94	128164.73	202618.47	0.57
33	P06396	GELS_HUMAN	Gelsolin	secreted	x					203446.28	190926.47	193045.77	200160.00	241074.69	146775.03	195904.71	0.15	311364.41	264604.19	346306.50	291620.66	326114.81	258141.36	299735.32	0.12	314876.56	292917.22	356668.44	370425.84	402272.91	294018.88	338529.97	0.13	248101.55	260477.19	311221.53	300261.38	293211.97	254252.00	279524.27	0.09
34	P02760	AMBP_HUMAN	Protein AMBP	secreted	x					191524.44	189318.72	198842.23	174188.22	211566.59	165443.92	188480.69	0.09	254834.98	223711.86	237497.47	205434.14	213563.20	218272.86	225552.42	0.08	290472.63	241300.03	227620.36	240125.78	321136.75	237764.38	259736.65	0.14	201312.03	229465.70	234458.86	222458.77	223221.88	246503.89	226236.85	0.07
35	P02753	RET4_HUMAN	Retinol-binding protein 4	secreted	x					242004.47	198464.25	185327.98	189278.39	173655.83	140872.00	188267.15	0.18	357111.25	273361.91	252041.52	243568.83	205010.53	254291.92	264230.66	0.19	305545.22	280619.75	238759.52	268891.97	215860.75	254357.55	260672.46	0.12	217923.25	245996.67	221540.33	224395.00	166706.47	229457.13	226669.81	0.15
36	P10909	CLUS_HUMAN	Clusterin	secreted	x					180937.58	165127.97	207150.78	189618.06	229061.31	145490.02	186248.12	0.16	276367.72	227805.53	266431.31	237859.69	232161.06	242912.50	247306.14	0.08	331450.84	262647.94	272910.03	303227.84	268514.69	231789.34	278423.46	0.12	214449.98	224455.63	216160.53	219104.84	199394.05	230114.11	217313.22	0.05
37	P25311	ZA2G_HUMAN	Zinc-alpha-2-glycoprotein	secreted	x					208755.20	185552.16	151517.34	197062.00	174411.55	160001.13	179549.90	0.12	280545.81	250327.77	219929.86	258337.86	205665.69	216004.19	238468.53	0.12	323065.59	250057.19	199621.20	283456.56	247489.13	237464.88	256859.09	0.16	216519.92	235295.44	181383.33	235516.42	195980.55	223482.81	214696.41	0.10
38	P01019	ANGT_HUMAN	Angiotensinogen	secreted	x					172311.14	187132.23	128661.73	163830.06	160633.92	257550.53	178353.27	0.24	322459.59	300949.88	194018.75	268088.97	231475.64	474659.38	298608.70	0.33	361355.56	340398.53	227648.86	290473.91	239011.56	557937.88	336137.72	0.36	272534.50	306230.69	227144.27	238274.77	215983.31	535088.94	299209.41	0.40
39	P0C0L4	CO4A_HUMAN	Complement C4-A	secreted	x					140483.55	141578.91	195363.73	222600.22	181448.86	99854.95	164489.54	0.27	131411.47	129898.20	187349.95	258365.09	139180.06	114979.22	160197.33	0.34	127984.59	109616.88	159335.13	245935.36	136800.42	95854.96	145922.22	0.37	144345.53	189211.69	210500.97	275695.22	189720.73	160275.77	194958.32	0.24
40	P01031	CO5_HUMAN	Complement C5	secreted	x					180381.00	182692.88	136376.61	150502.31	170588.70	165086.09	164271.27	0.11	274343.94	273332.22	316419.47	255928.27	259651.05	323289.84	283827.46	0.10	306614.91	301286.13	297862.75	270816.66	311159.34	325374.81	302185.77	0.06	201187.19	217900.28	249784.98	196998.61	221133.75	251756.38	223126.86	0.10
41	P04196	HRG_HUMAN	Histidine-rich glycoprotein	secreted	x					118481.19	178407.41	232806.38	114781.19	192846.83	94759.15	155347.02	0.35	161571.05	228270.08	251400.88	135711.75	195726.16	158715.63	188565.92	0.24	201319.55	282831.38	296027.84	195891.70	234776.22	193072.69	233986.56	0.20	117861.39	203909.19	195654.39	117387.80	145701.20	135363.23	152646.20	0.25
42	P19652	A1AG2_HUMAN	Alpha-1-acid glycoprotein 2	secreted	x					197835.28	113024.99	119912.66	204930.64	166827.50	126409.01	154823.35	0.26	166045.00	95764.39	103651.12	171176.53	180225.80	146780.03	143940.48	0.25	187913.39	142655.27	126808.39	272088.41	229089.48	208928.09	194580.44	0.28	160974.67	101142.63	126665.76	193463.55	163892.67	184747.95	155481.21	0.22
43	P02751	FINC_HUMAN	Fibronectin	secreted	x					229810.88	174718.98	112011.97	89230.21	112240.23	109347.37	144576.61	0.36	134914.98	114243.20	72024.39	58633.45	56694.67	73236.23	84674.49	0.38	643268.88	654998.50	387538.63	326917.13	451415.81	489912.69	492008.60	0.27	470258.41	601226.19	460240.69	476869.53	339421.47	529906.00	482988.71	0.18
44	P04003	C4BPA_HUMAN	C4b-binding protein alpha chain	secreted	x					146131.52	107171.98	111429.80	130396.19	139028.42	107096.00	123542.32	0.14	164701.38	99282.88	108331.73	133250.28	120403.17	118188.85	124026.38	0.19	226129.52	126935.98	142881.63	171932.61	197046.20	170897.66	172637.27	0.21	171337.30	141208.97	153696.38	169335.63	160973.61	182798.22	163225.02	0.09
45	P05546	HEP2_HUMAN	Heparin cofactor 2	not specified	x					126764.26	121221.07	87420.00	100557.55	112387.58	137818.19	114361.44	0.16	197060.05	154089.22	142913.78	135164.97	135070.91	208914.78	162202.28	0.20	219771.03	180622.58	165154.98	162451.56	163893.22	244391.63	189380.83	0.18	158179.98	143518.55	128671.90	133868.08	141688.55	212461.88	153064.82	0.20
46	P02649	APOE_HUMAN	Apolipoprotein E	secreted	x					135306.03	128231.03	91264.40	87353.80	150347.55	74034.13	111089.49	0.28	188062.20	166530.84	128718.67	124848.16	170564.73	116587.59	149218.70	0.20	197236.47	172814.36	117347.48	120829.34	192520.19	125658.72	154401.09	0.24	166003.81	169255.31	126074.41	121279.38	174813.20	126903.94	147388.34	0.17
47	P01834	IGKC_HUMAN	Ig kappa chain C region	not specified	x					73337.63	80994.29	199787.63	95326.25	96450.00	81239.22	105452.54	0.45	149203.83	95091.23	199822.06	158640.02	119087.25	157182.94	146504.73	0.25	230129.27	197576.02	252745.86	230676.81	209617.72	187334.73	218013.40	0.12	158402.09	135189.91	221876.02	134632.50	106487.22	132555.69	148190.57	0.27
48	P02656	APOC3_HUMAN	Apolipoprotein C-III	secreted	x					128326.38	110432.16	87930.59	113477.86	125946.30	64597.54	105118.47	0.23	356210.94	239383.83	212753.19	248715.22	250032.48	202348.45	251409.02	0.22	482493.78	316993.59	212101.30	316063.59	291391.94	247493.75	311089.66	0.30	261993.56	218625.44	178855.95	213984.52	241789.34	187059.92	217051.46	0.15
49	P08697	A2AP_HUMAN	Alpha-2-antiplasmin	secreted	x					131154.75	114134.87	79483.96	104610.92	94167.86	90881.72	102405.68	0.18	164026.80	146156.06	168650.39	133236.67	122357.81	167203.23	150271.83	0.13	161380.28	135754.23	154035.94	177300.78	173514.59	171420.09	162234.32	0.10	64952.28	65208.52	70511.03	60605.99	57083.46	64379.17	63790.07	0.07
50	P27169	PON1_HUMAN	Serum paraoxonase/arylesterase 1	secreted	x					85870.23	139520.67	126570.80	93899.32	81985.09	66310.87	99359.50	0.28	205413.84	262397.27	211260.66	209026.98	166022.03	166426.28	203679.63	0.18	1283621.75	1273877.88	1508991.69	822822.38	922062.38	741353.19	1000438.21	0.23	135846.83	217369.33	189310.19	142441.78	127979.26	141838.91	159131.05	0.22
51	P43652	AFAM_HUMAN	Afamin	secreted	x					116859.53	103631.31	88471.73	92778.35	86805.64	99678.70	98048.55	0.13	142651.83	136125.13	121019.06	91664.29	105537.31	137077.97	122345.93	0.17	186307.28	166701.55	125954.31	126634.47	130528.58	178919.28	152445.90	0.18	122828.92	119095.38	99263.36	78289.23	95556.63	123489.35	106737.15	0.17
52	P05090	APOD_HUMAN	Apolipoprotein D	secreted	x					90739.14	76000.34	85374.80	104456.90	131987.13	79652.66	94701.83	0.22	161766.66	147597.28	160338.59	166782.27	162947.28	139832.44	156544.09	0.07	149823.03	98714.28	120707.88	150332.38	192222.59	116485.73	138047.65	0.24	98795.06	88358.25	89921.81	112189.33	126045.20	94155.55	101577.54	0.15
53	P02743	SAMP_HUMAN	Serum amyloid P-component	secreted	x					80353.45	81875.17	110255.41	92770.74	98471.70	100111.40	93972.98	0.12	106495.88	92320.71	129143.35	97931.19	97703.18	117142.66	106789.49	0.13	151971.69	104768.28	133899.69	112538.96	130204.69	135812.38	128199.25	0.13	83809.56	86436.09	121070.90	95671.27	98008.85	125600.09	101766.13	0.17
54	P09871	C1S_HUMAN	Complement C1s subcomponent	not specified	x					75146.50	94992.18	77287.83	76304.63	100163.38	59870.31	80627.47	0.18	88003.16	110908.05	105033.97	81086.51	92430.42	84887.30	93725.07	0.11	92407.52	111006.63	109033.16	80874.20	86667.66	71469.56	91909.79	0.16	91276.23	124289.04	102440.31	92943.35	103803.61	87989.59	100457.75	0.13
55	Q96PD5	PGRP2_HUMAN	N-acetylmuramoyl-L-alanine amidase	secreted	x					87493.27	96612.54	64437.02	74523.54	88309.08	54745.75	77686.86	0.21	109535.55	123837.34	107054.85	97509.74	108925.58	79633.60	104416.11	0.14	93921.08	93828.21	86720.79	100505.02	105436.20	64398.12	90801.57	0.16	85254.06	99233.29	90159.87	82601.13	83109.11	64186.21	84090.61	0.14
56	P10643	CO7_HUMAN	Complement component C7	secreted	x					62896.84	89372.19	59773.19	67228.11	109966.86	63438.71	75445.91	0.27	89913.23	132717.63	108951.18	91146.84	123915.28	113500.16	110024.05	0.16	109729.59	137821.67	116790.00	118330.13	155906.58	130924.70	128237.09	0.13	79222.06	126672.71	107344.98	89439.82	134423.50	109842.77	107824.31	0.20
57	P00736	C1R_HUMAN	Complement C1r subcomponent	not specified	x					69369.78	90773.82	71487.39	71878.23	79640.77	55303.80	73075.63	0.16	116273.11	141671.91	142036.83	121100.15	129376.39	117530.19	127998.10	0.09	115344.26	148528.30	141726.83	146010.75	152904.61	129836.74	139058.58	0.10	111616.66	152342.23	136380.05	115094.54	120339.60	114666.06	125073.19	0.13
58	P02655	APOC2_HUMAN	Apolipoprotein C-II	secreted	x					100959.58	65832.88	80016.52	74993.87	64225.96	35381.92	70235.12	0.31	167690.25	95798.15	116890.46	106705.85	77666.12	85155.55	108317.73	0.30	135781.22	88554.34	92939.88	108144.92	58930.41	69292.90	92273.95	0.30	137992.66	100059.09	120368.15	128906.30	78444.63	89912.73	109280.59	0.21
59	P07358	CO8B_HUMAN	Complement component C8 beta chain	secreted	x					62942.85	79471.31	54995.77	63364.16	69134.45	54890.26	64133.13	0.14	44617.39	62679.68	67311.94	50259.99	37495.31	51342.37	52284.45	0.21	194711.47	252552.05	181338.33	207212.88	198697.11	185827.59	203389.90	0.13	65962.82	87670.40	87049.43	76523.41	77828.48	72285.74	77886.71	0.11
60	P01857	IGHG1_HUMAN	Ig gamma-1 chain C region	secreted	x					44522.21	57949.68	131486.23	41589.05	53930.14	45033.76	62418.51	0.55	52356.00	34865.90	98807.86	47668.84	60631.81	54434.97	58127.56	0.37	118124.12	111578.43	174451.95	96896.29	97532.55	99063.68	116274.50	0.26	64685.97	93733.79	146658.41	57482.90	60832.05	61010.18	80733.88	0.43
61	P51884	LUM_HUMAN	Lumican	secreted	x					51990.88	55054.25	54841.59	47412.91	64255.38	41465.30	52503.38	0.15	72326.71	68748.78	75975.84	62905.61	77103.12	63792.30	70092.79	0.07	90835.53	85900.51	79047.50	80517.36	94960.05	73337.39	84099.74	0.10	46969.80	49814.44	48386.36	42938.50	47345.18	46418.93	46978.87	0.05
62	P02747	C1QC_HUMAN	Complement C1q subcomponent subunit C	secreted	x					49848.96	66436.28	45984.20	47127.56	59662.05	43081.35	52023.40	0.17	60870.74	69027.66	54542.00	50049.58	54710.96	44684.61	55647.59	0.15	96271.37	84572.83	68151.60	71265.30	97935.81	72005.02	81700.32	0.16	50768.80	68227.22	56519.05	57351.02	71793.61	56075.98	60122.61	0.13
63	P0C0L5	CO4B_HUMAN	Complement C4-B	secreted	x					40840.60	56889.30	84271.18	46132.47	55895.39	45166.72	51532.61	0.17	85683.95	91186.88	95003.34	82694.78	70000.92	100984.86	87592.45	0.12	98000.56	103939.90	98445.21	95880.64	75924.94	123956.92	99358.03	0.16	58859.86	82391.45	84707.76	62704.05	63101.79	94297.50	74343.73	0.20
64	P01871	IGHM_HUMAN	Ig mu chain C region	secreted	x					38793.50	64757.53	42487.82	37126.91	66581.67	57516.42	51210.64	0.26	88341.56	113009.23	96985.16	62567.74	71316.64	79552.50	85295.47	0.21	39389.01	46173.16	41482.93	27817.45	32262.82	33822.47	36824.64	0.18	38446.30	55979.71	45899.76	30992.98	34126.18	40470.42	40985.89	0.22
65	Q3V6T2	GRDN_HUMAN	Girdin	leakage	x					50502.17	50747.35	45349.48	41755.91	72457.54	35350.89	49360.56	0.26	11265.57	14647.29	12455.39	12513.40	14501.84	15816.51	13167.00	0.10	10826.29	9090.12	12785.08	15309.09	19759.39	13140.34	13186.72	0.28	4869.07	4201.51	5204.81	4891.38	3953.37	4784.61	4650.90	0.10
66	P13671	CO6_HUMAN	Complement component C6	secreted	x					31401.55	62843.82	51777.65	34756.47	70647.80	41774.86	48867.03	0.32	39942.84	81393.31	78622.16	46822.80	68582.24	67399.43	63793.80	0.26	82591.16	125829.09	108613.64	89412.04	118133.59	107110.58	105281.68	0.16	67065.25	124696.91	110742.27	80920.78	126252.13	102849.07	102087.74	0.23
67	P03952	KLKB1_HUMAN	Plasma kallikrein	secreted	x					58847.52	49739.89	32956.46	43818.29	46060.30	43555.52	47201.98	0.18	89151.26	75321.49	64086.49	61052.86	86436.16	72012.60	71678.49	0.14	112589.27	73009.84	80513.57	78564.18	101069.60	85012.39	88459.81	0.17	90587.93	84305.64	82647.98	80209.41	82687.34	94646.13	84302.09	0.06
68	P07360	CO8G_HUMAN	Complement component C8 gamma chain	secreted	x					45607.89	47974.58	51967.82	46631.48	43626.51	35252.79	45176.85	0.12	48424.04	49846.21	52631.62	49683.02	35492.07	40535.79	45872.13	0.14	59649.45	52424.11	65469.08	66527.75	49408.69	62061.65	59226.79	0.12	45069.38	47686.59	51189.10	47548.20	34829.55	47165.82	45581.44	0.12
69	P36955	PEDF_HUMAN	Pigment epithelium-derived factor	secreted	x					60533.83	55304.46	35727.59	50855.67	45276.10	22025.39	44953.84	0.31	117623.27	86358.02	72359.91	80773.34	65730.17	45560.69	78067.57	0.31	126484.40	91563.09	70572.53	92387.33	70273.99	46514.18	82965.92	0.33	91162.15	81931.73	64856.50	72455.39	60817.16	39440.03	68443.83	0.26
70	P01876	IGHA1_HUMAN	Ig alpha-1 chain C region	not specified	x					41641.50	39876.94	60989.62	43867.54	43799.05	36358.22	44422.15	0.19	40630.92	12946.42	20743.57	27163.20	13391.84	25592.54	23411.42	0.44	66715.59	38321.98	63181.03	63754.04	27240.76	51438.35	51775.29	0.31	21968.49	16168.25	31079.74	17146.49	11824.30	15123.85	18885.19	0.36
71	P02750	A2GL_HUMAN	Leucine-rich alpha-2-glycoprotein	secreted	x					28293.54	36137.70	25843.63	49825.71	76118.84	41002.93	42870.39	0.43	37198.39	50269.82	46521.18	64433.98	88758.37	71849.28	59838.50	0.32	40899.85	52968.32	41563.30	80664.16	91155.84	71213.52	62944.16	0.34	31316.51	42188.18	35719.87	62432.01	74119.77	63403.05	51529.90	0.34
72	P35542	SAA4_HUMAN	Serum amyloid A-4 protein	secreted	x					29292.27	49166.52	33932.61	37521.22	68879.27	28547.86	41223.29	0.38	84605.42	94725.86	82622.63	68047.67	73218.23	56842.98	76677.13	0.18	59089.99	42050.96	34762.23	52448.41	43579.31	32158.17	44014.85	0.23	99314.82	111574.38	82780.72	86623.73	100829.41	64417.28	90923.39	0.18
73	P02746	C1QB_HUMAN	Complement C1q subcomponent subunit B	secreted	x					41915.07	55140.45	29765.03	38511.96	46999.83	32051.12	41063.91	0.24	77609.77	90349.44	79279.49	57118.00	76451.95	65374.26	74363.82	0.16	77631.08	77011.01	67618.98	70366.48	80254.25	64682.91	72927.45	0.09	47102.79	64123.66	55491.68	50062.49	57721.84	51035.45	54256.32	0.11
74	P05156	CFAI_HUMAN	Complement factor I	secreted	x					44473.06	38870.71	40109.10	38598.06	45826.74	37438.54	40886.04	0.08	61374.39	55168.07	66432.27	52209.74	55991.09	64258.84	59239.07	0.09	83982.65	75017.09	77562.07	81913.66	88228.73	82719.12	81570.55	0.06	65952.39	67132.07	73221.77	66984.59	68394.10	73822.77	69251.28	0.05
75	P35858	ALS_HUMAN	Insulin-like growth factor-binding protein complex acid labile subunit	secreted	x					39410.42	47437.65	28958.99	42258.41	41237.11	40191.05	39915.61	0.15	52605.04	58295.63	43778.43	52338.13	42524.04	61378.91	51820.03	0.15	45014.68	48272.64	33946.17	43867.59	39816.52	51899.91	43802.92	0.14	30654.08	39853.18	26223.27	31169.82	27996.24	39763.14	32609.96	0.18
76	P02654	APOC1_HUMAN	Apolipoprotein C-I	secreted	x					57467.56	42564.35	39316.12	41686.65	39889.51	17989.37	39818.93	0.32	84297.77	56179.82	60684.63	70743.59	66150.81	44408.95	63744.26	0.21	153238.06	67904.00	60184.45	62995.25	107954.75	59734.29	85335.13	0.45	76656.78	65019.21	71884.71	57203.48	75153.61	52236.40	66359.03	0.15
77	P05543	THBG_HUMAN	Thyroxine-binding globulin	secreted	x					25749.46	34987.73	36855.36	32129.55	54094.11	47257.21	38512.24	0.27	42188.45	45759.72	50948.00	45038.21	64777.87	79078.93	54631.86	0.26	42792.13	59507.27	60226.29	53386.71	73467.79	92461.51	63640.28	0.27	37963.00	50231.86	51183.59	37213.27	55208.97	84322.85	52687.26	0.33
78	P07357	CO8A_HUMAN	Complement component C8 alpha chain	secreted	x					31076.74	38004.29	57613.69	34434.14	38456.52	24625.53	37368.48	0.30	32577.48	40726.23	38002.82	31442.61	25072.19	32159.12	33330.08	0.16	32453.85	38182.00	40572.75	41730.52	28263.39	35046.44	36041.49	0.14	28069.92	33651.93	34864.33	31333.08	22001.27	29250.52	29861.84	0.15
79	Q15149	PLEC_HUMAN	Plectin	leakage	x					30777.21	35875.28	33133.22	39873.18	38609.38	31432.94	34950.20	0.11	47907.31	52669.37	68349.66	61289.41	51216.76	65107.50	57756.67	0.14	40082.64	35632.59	35521.74	46208.66	43206.69	43617.27	40711.60	0.11	43577.34	53799.91	49790.22	45625.02	43669.98	43115.75	46596.37	0.09
80	P08185	CBG_HUMAN	Corticosteroid-binding globulin	secreted	x					31821.48	32461.65	30838.74	30671.99	32845.77	46255.52	34149.19	0.18	51802.61	39344.49	53225.82	44169.12	48727.02	86494.57	53960.61	0.31	71306.39	60405.09	72064.65	63535.32	62868.95	114131.98	74052.06	0.27	45857.65	42712.14	55733.36	43123.98	42051.70	88472.99	52991.97	0.34
81	P07225	PROS_HUMAN	Vitamin K-dependent protein S	secreted	x					43561.46	29557.28	23246.67	36134.20	29585.46	32197.98	32380.51	0.22	66175.86	51314.74	56080.22	53309.06	46889.09	45147.78	53152.79	0.14	84567.86	60549.85	84326.60	73195.73	76550.00	82279.13	76911.53	0.12	69093.32	48262.45	59038.93	55266.64	52645.26	59417.30	57287.32	0.12
82	P22792	CPN2_HUMAN	Carboxypeptidase N subunit 2	secreted	x					35637.09	32043.16	23495.47	37533.95	32595.62	26842.53	31357.97	0.17	59776.67	46464.31	47101.91	54718.29	44635.32	49756.61	50408.85	0.11	70538.37	51899.11	45384.70	67579.71	57843.27	59552.43	58799.60	0.16	74007.63	64456.27	61511.21	68248.05	54092.39	68402.59	65119.69	0.11
83	Q5VT06	CE350_HUMAN	Centrosome-associated protein 350	leakage	x					31112.42	31499.31	40511.09	29857.06	30022.68	23897.22	31149.96	0.17	100042.94	103063.96	119855.67	177104.06	121120.76	123225.65	124068.84	0.22	48550.26	49167.87	43347.67	65686.63	61692.34	72871.77	56886.09	0.20	82639.59	117220.12	89519.50	131880.94	106025.46	126905.19	109031.80	0.18
84	P29622	KAIN_HUMAN	Kallistatin	secreted	x					35908.42	30960.03	27053.55	29666.53	32587.46	28910.64	30847.77	0.10	55256.33	45277.94	49122.43	44646.11	45659.50	52614.75	48762.84	0.09	60653.25	49026.15	49287.27	54323.14	52982.05	60854.90	54521.13	0.10	45306.83	43046.88	40060.20	40520.35	37521.71	48066.60	42420.46	0.09
85	Q8TD57	DYH3_HUMAN	Dynein heavy chain 3, axonemal	leakage	x					34369.89	32587.56	20231.25	32672.08	29944.09	29357.21	29860.35	0.17	47644.66	39202.71	46860.50	53599.63	45753.74	43502.43	46093.94	0.10	80482.27	64408.87	68740.33	80784.52	64471.48	61297.63	70030.85	0.12	21761.84	29050.32	29422.33	28140.53	31311.14	26991.78	27779.66	0.12
86	Q02224	CENPE_HUMAN	Centromere-associated protein E	leakage	x					29461.80	30018.16	23854.54	28406.81	33179.93	30704.27	29270.92	0.11	25929.18	29313.66	31902.88	34234.98	29784.02	30356.80	30253.59	0.09	11678.10	8433.08	7702.46	8717.98	11027.65	9563.49	9520.46	0.16	31144.06	38536.36	33769.04	38446.05	41197.83	38284.76	36896.35	0.10
87	Q7Z5M8	AB12B_HUMAN	Protein ABHD12B	not specified	x					30464.14	30316.33	28298.82	24342.86	30999.28	23213.84	27939.21	0.12	8037.82	9834.86	13097.79	11444.96	12971.23	12029.80	11371.08	0.17	16022.83	15892.44	13396.97	12531.77	16127.33	12439.53	14401.81	0.12	13631.51	12917.60	14133.72	9730.94	23507.03	8657.46	13763.04	0.38
88	P00751	CO2_HUMAN	Complement C2	secreted	x					28374.63	36783.40	21602.01	23020.63	29309.04	22392.37	26913.68	0.22	58594.59	62519.65	63577.26	51888.78	57370.85	64294.95	62539.56	0.11	59885.91	68406.22	54104.54	64760.16	57773.88	60719.26	60941.66	0.08	45311.25	53567.51	47136.72	41391.39	51985.79	48195.95	48065.59	0.14
89	O95445	APOM_HUMAN	Apolipoprotein M	secreted	x					28146.80	26660.57	17300.82	22877.82	28044.54	17747.00	23462.93	0.21	27386.60	24499.91	23661.75	21260.87	24652.85	18864.58	23387.76	0.13	45922.69	35676.71	37312.68	30730.49	55971.11	32915.62	40354.87	0.22	32019.25	30567.30	34025.69	32994.11	35568.00	28557.42	32305.43	0.08
90	Q86TB3	ALPK2_HUMAN	Alpha-protein kinase 2	not specified	x					18963.20	27168.90	16723.50	21588.76	22942.16	24802.37	22028.15	0.17	7498.23	7824.78	5641.34	9395.15	6187.39	9525.66	7678.76	0.21	75173.91	52731.22	65205.23	57941.85	84445.15	56203.02	65283.40	0.19	38442.08	42623.04	34920.72	40816.08	37177.90	40035.77	39002.60	0.08
91	P00748	FA12_HUMAN	Coagulation factor XII	secreted	x					28778.16	28342.11	15337.74	19503.63	19599.63	17115.79	21446.18	0.27	39699.97	43214.09	42460.26	34169.41	34552.53	33233.78	37888.34	0.12	53232.89	38365.93	52445.98	51180.11	68404.99	45455.78	51505.95	0.19	31592.34	32857.53	32749.12	32281.63	27082.47	26862.99	30571.01	0.09
92	Q8NFC6	BD1L1_HUMAN	Biorientation of chromosomes in cell division protein 1-like 1	leakage	x					16664.35	18746.70	32681.70	17001.86	25257.06	14788.78	20856.74	0.33	3836.28	3632.11	3411.05	4843.46	6060.27	3321.42	4184.10	0.26	6230.29	3738.01	5406.16	4844.12	5730.88	3880.76	4971.71	0.20	2014.24	2091.56	1778.46	2215.08	2826.66	1849.56	2129.26	0.18
93	P00742	FA10_HUMAN	Coagulation factor X	secreted	x					22422.43	19449.44	15739.19	20072.14	23986.39	16282.44	19658.67	0.17	18585.35	17924.99	21172.58	17093.05	18718.20	20627.45	19036.94	0.08	29108.72	14319.79	22488.98	23362.55	31581.87	21970.49	23805.40	0.25	17915.67	14347.13	17588.34	19349.58	17571.24	20882.19	17942.36	0.12
94	P08519	APOA_HUMAN	Apolipoprotein	secreted	x					775.03	1326.96	120.95	76940.86	568.75	29718.39	18241.82	1.70	4321.40	4828.00	5076.55	112123.33	3250.39	55241.53	30806.87	1.45	11210.56	12711.51	12862.77	151952.34	13114.86	71112.18	45494.04	1.26	835.37	425.83	663.93	98053.73	485.26	40912.90	23562.84	1.69
95	P06276	CHLE_HUMAN	Cholinesterase	secreted	x					9914.12	13669.82	20968.66	25593.44	27199.20	11586.25	18146.34	0.41	10800.37	8405.16	9576.12	8405.23	9963.92	9690.16	9485.16	0.10	19111.59	15041.66	16131.53	14044.09	15889.66	15149.49	15969.92	0.11	5036.90	5451.62	5646.60	5946.77	4690.85	5446.09	5386.60	0.17
96	Q06033	ITIH3_HUMAN	Inter-alpha-trypsin inhibitor heavy chain H3	secreted	x					18180.06	19101.98	13528.29	16340.88	26096.69	15538.02	18130.99	0.24	27229.07	28374.40	38891.20	28646.96	42811.35	30528.60	32746.93	0.20	200157.14	103064.19	178033.05	105713.73	160170.56	154493.59	150272.04	0.26	22652.51	26450.90	37814.90	26650.94	36833.58	26080.89	29423.61	0.21
97	P00738	HPT_HUMAN	Haptoglobin	secreted	x					24786.44	19941.94	14971.63	18527.00	13687.48	16261.54	18029.34	0.22	47586.16	37150.93	37423.18	45585.86	37476.18	42095.73	41219.67	0.11	65466.56	50979.72	57743.14	60536.69	43651.14	56932.23	56161.72	0.13	43695.35	41138.57	38294.72	38573.85	32652.89	54904.18	41259.85	0.18
98	P05160	F13B_HUMAN	Coagulation factor XIII B chain	secreted	x					24809.66	17697.27	9124.57	16603.02	19780.90	17927.83	17657.21	0.29	30358.10	24419.26	25900.73	22942.77	24734.62	25382.22	25622.95	0.10	23753.73	20667.26	22987.47	27412.71	30027.72	20011.10	24143.33	0.16	27431.62	21600.37	19796.87	22918.27	22887.16	27164.72	23633.23	0.13
99	P05452	TETN_HUMAN	Tetranectin	secreted	x					18897.94	17162.54	13839.79	14458.65	22563.40	12491.49	16568.97	0.23	40993.93	32718.11	30851.34	32398.03	38770.84	27649.34	34080.26	0.16	102264.88	73244.85	110779.48	88098.15	104567.20	93677.51	110438.68	0.39	36817.02	34680.08	33906.09	33011.11	40221.15	31528.37	35027.30	0.09
100	O75533	SF3B1_HUMAN	Splicing factor 3B subunit 1	leakage	x					17062.07	16810.46	13653.47	15497.95	12526.21	18709.28	15709.91	0.15	89836.16	89694.07	104156.27	93041.98	112503.38	86666.25	95524.68	0.11	20377.79	22534.19	24892.31	26598.47	29555.04	19518.02	23904.30	0.16	10458.03	8390.38	7336.78	7682.30	10150.20	9527.41	8924.18	0.13
101	P80108	PHLD_HUMAN	Phosphatidylinositol-glycan-specific phospholipase D	secreted	x					21526.34	17077.72	11914.91	16448.47	9724.90	11448.53	14690.14	0.30	25986.13	17643.04	23311.82	20728.91	13996.78	16755.50	19737.03	0.23	26252.89	15848.91	19271.41	22334.54	23523.03	20015.98	21207.79	0.17	18201.77	14173.34	15482.63	16771.27	12093.19	14507.11	15204.89	0.14
102	P01009	A1AT_HUMAN	Alpha-1-antitrypsin	secreted	x					13265.98	12195.54	27632.64	12389.13	13366.91	6709.61	14259.97	0.50	14560.93	15154.61	34390.72	22657.49	30048.60	21211.04	22993.73	0.34	27879.37	12322.00	30480.40	30543.49	21460.33	14439.43	22854.17	0.35	35170.42	28318.75	49641.80	31943.52	21531.56	15576.70	29692.67	0.40
103	Q7L5N1	CSN6_HUMAN	COP9 signalosome complex subunit 6	leakage	x					14954.99	14797.11	8988.39	14505.41	14683.32	16784.72	14118.99	0.19	37869.12	37154.56	33992.12	52191.17	40490.46	42020.27	40619.62	0.16	50210.08	52232.96	52579.12	55313.03	57809.74	60484.27	54771.53	0.07	34832.84	47339.05	43947.32	54987.74	44656.36	60457.58	47703.48	0.19
104	O75882	ATRN_HUMAN	Attractin	secreted	x					13344.71	13210.86	10002.04	15789.23	17433.10	11494.69	13545.77	0.20	20563.70	25037.12	24568.22	20220.99	23304.75	23866.18	22926.83	0.09	30393.41	29817.59	28204.22	36163.34	32277.27	33739.43	31750.88	0.09	21021.53	22292.93	21908.08	23629.87	24332.89	22549.36	22694.11	0.05
105	Q8IVF4	DYH10_HUMAN	Dynein heavy chain 10, axonemal	leakage	x					12634.24	14536.17	11344.24	15087.19	13455.63	12859.69	13319.52	0.10	869623.06	720309.25	640406.31	723205.94	927950.94	560449.00	740775.75	0.19	109655.92	121872.19	104272.00	108014.88	90956.19	99426.76	105699.66	0.10	88552.69	109670.45	101527.61	91708.88	104974.39	91550.02	97997.34	0.09
106	P01859	IGHG2_HUMAN	Ig gamma-2 chain C region	secreted	x					16235.24	5408.72	20171.12	20239.98	7605.10	7528.91	12864.85	0.53	18220.03	8403.53	32685.76	36694.98	14964.07	30354.46	23553.81	0.48	39346.78	13630.65	31281.80	51583.81	11590.89	34543.46	30329.57	0.51	30890.52	10195.06	28393.19	25660.24	8746.44	17424.98	20218.41	0.47
107	P23142	FBLN1_HUMAN	Fibulin-1	secreted	x					14427.30	15438.88	8304.35	10242.04	11770.21	14435.25	12436.34	0.23	4651.69	5637.74	4555.73	5277.20	3156.43	4480.82	4626.60	0.18	3598.81	2777.98	2777.55	2410.38	3579.30	2815.04	2993.18	0.16	3323.75	3492.66	2949.05	3843.23	3263.04	4368.07	3539.97	0.14
108	Q13790	APOF_HUMAN	Apolipoprotein F	secreted	x					15539.15	13688.13	6284.54	13007.06	11693.28	10178.70	11731.81	0.28	13020.22	14326.60	16992.92	14185.57	13641.46	12379.25	14090.67	0.11	11969.12	12951.18	13173.27	13596.47	11990.62	11261.22	12474.36	0.07	22900.94	20900.87	31471.41	23328.66	20435.05	24688.34	23954.21	0.17
109	P00739	HPTR_HUMAN	Haptoglobin-related protein	secreted	x					5636.60	12495.35	14245.04	10297.75	12893.41	9200.79	10794.82	0.29	12960.10	20424.84	14344.67	17426.53	16581.91	21619.64	17226.28	0.20	17683.61	31725.89	24599.56	23756.32	22697.20	31638.65	25350.21	0.22	17654.75	30155.88	23586.58	22748.39	20569.46	34613.55	24888.10	0.25
110	P02745	C1QA_HUMAN	Complement C1q subcomponent subunit A	secreted	x					12477.59	14499.65	8375.14	9567.14	10186.17	8837.55	10657.21	0.22	11813.49	13288.81	15090.11	12618.54	9654.54	12096.58	12427.01	0.14	13597.72	16163.18	14018.74	16704.74	13855.48	15855.89	14957.69	0.09	12953.19	12357.74	17263.27	10330.38	12067.47	10475.72	12574.63	0.20
111	P20742	PZP_HUMAN	Pregnancy zone protein	secreted	x					9686.59	9520.89	8254.66	11215.07	11116.15	10194.40	9997.96	0.11	28871.59	20696.78	30589.46	20246.96	20747.47	23436.72	24098.16	0.19	1874.15	1810.32	2863.86	2336.05	2221.47	3220.76	2387.77	0.23	2121.32	3219.94	2795.95	3737.06	1727.11	3586.62	2864.67	0.28
112	P01860	IGHG3_HUMAN	Ig gamma-3 chain C region	secreted	x					12259.14	11382.25	7242.11	8258.88	10329.93	9542.12	9835.74	0.19	10999.16	11245.73	9563.94	6341.41	8135.46	7222.76	8918.08	0.23	14380.06	14187.25	13257.12	9671.38	10508.30	13106.00	12526.02	0.15	20089.06	19624.60	19037.95	15056.50	16226.83	17068.05	17882.84	0.12
113	P27918	PROP_HUMAN	Properdin	secreted	x					9537.26	9426.69	5553.92	9364.11	11891.23	9334.15	9184.56	0.22	3462.80	5096.73	3992.47	4667.99	4098.50	5471.35	4464.97	0.17	25751.07	27423.52	20787.03	25374.86	23702.86	26812.72	24975.34	0.10	10088.56	11224.10	9818.26	9574.45	8934.89	7083.36	9619.26	0.17

Supplementary material Overview on all sample collection methods relatively quantified proteins after depletion

										EDTA plasma samples								heparin plasma samples								citrate plasma samples								serum samples							
Nr	Accession	Entry name	Protein names	secreted/ leakage	core set	unique EDTA	unique heparin	unique citrate	unique serum	EDTA_1	EDTA_2	EDTA_3	EDTA_4	EDTA_5	EDTA_6	Mean abundance	CV	heparin_1	heparin_2	heparin_3	heparin_4	heparin_5	heparin_6	Mean abundance	CV	citrate_1	citrate_2	citrate_3	citrate_4	citrate_5	citrate_6	Mean abundance	CV	serum_1	serum_2	serum_3	serum_4	serum_5	serum_6	Mean abundance	CV
114	P04180	LCAT_HUMAN	Phosphatidylcholine-sterol acyltransferase	secreted	x					7956.95	9706.97	6839.78	10623.64	11346.25	8258.07	9121.94	0.19	9136.67	8044.83	7078.36	6979.39	7225.50	6668.74	7522.25	0.12	20863.15	25941.91	19414.19	22626.31	18778.08	18554.24	21029.65	0.14	6942.22	7039.52	5763.31	6535.85	6978.24	7108.73	6727.98	0.08
115	Q96IY4	CBPB2_HUMAN	Carboxypeptidase B2	secreted	x					8681.40	9196.20	6763.96	8331.58	8851.74	9139.07	8493.99	0.11	12754.80	12085.89	12491.82	9894.48	9611.78	13392.53	11705.22	0.13	20242.78	17146.15	15999.66	15978.85	21471.97	22248.81	18848.04	0.15	9527.41	10149.75	11094.70	9338.44	8874.66	13371.34	10392.72	0.16
116	P22352	GPX3_HUMAN	Glutathione peroxidase 3	secreted	x					8744.07	9765.40	5912.68	8065.11	9541.50	8680.95	8451.62	0.16	14912.96	14800.88	19122.86	12946.68	17231.08	16218.53	15872.16	0.14	13313.99	11487.55	15046.81	15735.77	17843.54	16099.39	14921.17	0.15	9952.82	10287.41	11467.34	10823.04	11797.58	11199.62	10921.30	0.06
117	Q04756	HGFA_HUMAN	Hepatocyte growth factor activator	secreted	x					10591.81	9166.38	3334.14	7077.66	11569.90	7803.05	8257.16	0.36	11572.29	9410.43	8964.15	5408.94	8974.88	8943.58	8879.04	0.22	12847.14	11808.77	10402.45	10259.61	16501.38	10816.74	12106.01	0.20	14267.63	14199.54	11256.52	9168.58	16539.40	11019.57	12741.87	0.21
118	P15169	CBPN_HUMAN	Carboxypeptidase N catalytic chain	secreted	x					9655.93	9927.24	3925.01	8985.29	8560.47	6082.27	7856.03	0.30	11379.61	12601.28	9547.79	10943.00	10694.55	11332.56	11083.13	0.09	11054.77	11293.43	11672.25	18319.58	13022.12	11034.44	12732.76	0.22	11948.33	11610.10	8217.09	11982.15	8677.52	10086.30	10420.25	0.16
119	O75636	FCN3_HUMAN	Ficolin-3	secreted	x					11483.60	5214.39	8462.32	8931.56	6684.34	6121.85	7816.34	0.29	23670.66	11038.11	17632.97	21680.49	15340.62	16693.62	17676.08	0.26	26454.19	14185.43	26724.64	26631.20	17045.13	17354.53	21399.19	0.27	15599.54	5640.94	15293.10	13987.24	8018.99	9224.25	11294.01	0.37
120	Q14520	HABP2_HUMAN	Hyaluronan-binding protein 2	secreted	x					8521.20	9237.24	4978.52	6932.49	5946.60	10846.69	7743.79	0.28	18117.10	19293.85	17786.33	14232.81	11243.18	21475.13	17024.73	0.22	15518.94	18887.96	19527.02	18554.12	16505.49	29664.63	19776.36	0.26	12756.12	13830.43	14745.34	9891.74	9164.58	16457.90	12807.69	0.22
121	Q15848	ADIPO_HUMAN	Adiponectin	secreted	x					9003.71	8581.82	4182.63	5908.42	10294.70	7168.31	7523.27	0.30	3758.48	2766.19	4176.79	1541.87	4045.90	3422.74	3285.33	0.30	93.49	71.46	1584.21	1944.00	2045.49	805.68	1090.72	0.82	8659.88	7848.78	7688.23	6049.87	9527.40	7088.39	7810.42	0.15
122	Q99698	LYST_HUMAN	Lysosomal-trafficking regulator	leakage	x					8169.67	8151.59	6956.24	5715.20	10554.11	5590.25	7522.84	0.25	128235.81	101756.84	126132.28	179741.64	165811.81	174131.42	145968.30	0.22	23969.27	19750.43	17435.07	21660.05	30272.39	22886.26	22662.24	0.19	6827.19	5690.67	8903.05	5296.04	9881.75	5657.49	7042.70	0.27
123	P43251	BTD_HUMAN	Biotinidase	secreted	x					8490.01	7163.62	8578.92	6918.60	6301.66	6105.92	7259.79	0.15	6959.66	5804.43	8712.50	6386.93	7279.23	8417.83	7260.10	0.16	13106.35	11670.34	16867.39	15168.00	17399.38	16468.69	15113.36	0.15	15948.44	20690.05	21236.55	21331.62	21253.14	21539.34	20333.19	0.11
124	Q96KN2	CNDP1_HUMAN	Beta-Ala-His dipeptidase	secreted	x					7433.52	7639.03	7698.21	7656.07	5955.61	7036.24	7236.45	0.09	25455.31	20530.16	28692.79	27016.63	21696.09	22516.21	24317.86	0.13	12307.69	11727.74	13332.51	12981.48	11135.96	12232.38	12286.29	0.07	14250.15	14531.73	17622.21	14619.88	11338.28	14754.46	14519.45	0.14
125	P20851	C4BPB_HUMAN	C4b-binding protein beta chain	secreted	x					8757.44	6613.08	5429.59	7675.04	7809.60	6121.53	7067.71	0.17	11694.62	8800.33	10496.67	9637.57	9841.57	9065.31	9922.68	0.11	20590.00	15342.11	15504.96	18203.27	19419.18	17368.21	17737.96	0.12	16263.02	13939.80	15156.66	14443.97	17602.08	14241.07	15274.43	0.09
126	P01861	IGHG4_HUMAN	Ig gamma-4 chain C region	secreted	x					6514.05	5396.45	8601.62	5938.63	5638.10	5912.37	6333.54	0.19	10755.65	9096.78	15425.38	7458.64	11045.24	11572.54	10892.37	0.25	7565.24	5504.07	11395.84	9838.35	9539.77	9932.86	8962.69	0.23	5315.90	5289.10	8130.09	5675.07	5644.66	5010.08	5844.15	0.20
127	P48740	MASP1_HUMAN	Mannan-binding lectin serine protease 1	secreted	x					8882.48	7001.68	2342.10	6050.72	5189.29	8345.59	6301.97	0.38	11282.64	12086.33	16437.51	10762.68	10037.01	10824.11	11905.05	0.19	7435.31	5300.21	10544.05	9910.65	6006.28	7786.92	7830.57	0.27	39219.46	43642.99	33526.02	38380.97	45435.11	41977.16	40363.62	0.11
128	Q16610	ECM1_HUMAN	Extracellular matrix protein 1	secreted	x					8707.60	6151.32	3366.72	7197.11	5088.55	6069.98	6096.88	0.30	13560.91	12905.38	14198.35	20878.41	15040.80	13142.39	14954.37	0.20	10247.43	8167.39	14861.74	15402.48	14846.81	12194.45	12620.05	0.23	4991.64	5209.49	6550.24	6648.90	4048.85	5450.08	5483.20	0.18
129	P00740	FA9_HUMAN	Coagulation factor IX	secreted	x					7363.59	6754.33	3255.71	5501.57	6444.41	6226.93	5924.42	0.24	14719.25	21383.00	19932.64	16084.52	16978.52	16500.38	17599.72	0.14	9793.63	17002.40	16016.18	17250.35	19402.73	14087.03	15592.05	0.21	14667.90	18349.62	19046.33	17907.50	21496.67	17943.44	18235.25	0.12
130	P04278	SHBG_HUMAN	Sex hormone-binding globulin	secreted	x					4484.65	6720.90	2706.07	3211.38	10565.20	6732.20	5736.73	0.51	8491.11	12566.20	10635.85	7134.13	19033.54	17773.00	12605.64	0.39	8271.42	14165.62	12252.63	11944.26	22461.04	22565.01	15276.66	0.39	7748.35	11505.73	8692.77	6509.60	14756.71	15711.88	10820.84	0.35
131	Q6KB66	K2C80_HUMAN	Keratin, type II cytoskeletal 80	not specified	x					2718.44	3561.10	9962.89	3519.54	7740.17	2273.76	4962.65	0.63	628.05	685.73	827.53	435.91	639.74	652.99	644.99	0.19	300549.34	260360.17	177391.70	231245.50	302198.19	220268.67	248668.93	0.20	2319.60	3818.91	2461.87	5799.39	8330.36	3024.48	4292.43	0.55
132	P36980	FHR2_HUMAN	Complement factor H-related protein 2	secreted	x					3779.74	4985.81	3928.37	4144.47	5687.59	6534.72	4843.45	0.23	3159.39	4065.20	5197.12	2350.12	3841.57	5713.64	4054.51	0.31	4480.29	3201.61	5110.86	3985.95	6194.66	6234.40	4867.96	0.25	3719.42	5702.48	6330.29	5396.49	5902.88	8610.41	5943.66	0.27
133	O14791	APOL1_HUMAN	Apolipoprotein L1	secreted	x					5088.60	7295.23	2370.54	4059.14	5072.92	5120.98	4834.57	0.33	6316.49	9271.08	5626.26	5622.22	7348.18	8297.39	7080.27	0.21	6657.12	10153.84	6259.83	9584.82	8770.32	11443.89	8811.64	0.23	7407.72	8844.95	5779.26	6649.49	7393.06	9364.90	7573.23	0.18
134	O43866	CD5L_HUMAN	CD5 antigen-like	secreted	x					4399.22	7396.07	3000.50	4187.69	5237.73	3716.78	4656.33	0.33	7366.51	11450.70	8744.58	7584.50	7885.98	9172.88	8700.86	0.17	6445.61	9306.93	7297.44	7660.65	9062.33	7610.63	7897.27	0.14	5368.23	8322.81	6023.95	5175.82	5414.83	5783.02	6014.77	0.19
135	Q9UGM5	FETUB_HUMAN	Fetuin-B	secreted	x					4669.72	4269.65	2240.04	3457.16	5292.08	8005.58	4655.70	0.42	10711.67	10137.07	10625.47	8800.32	10961.87	19887.85	11854.04	0.34	6854.78	5929.29	7378.02	7545.32	11655.92	18497.10	9643.41	0.49	6340.86	5342.94	6405.73	5115.65	8104.74	17595.53	8150.91	0.58
136	Q03591	FHR1_HUMAN	Complement factor H-related protein 1	secreted	x					316.89	5873.34	7562.33	224.63	7390.01	5291.65	4443.14	0.75	151.12	6615.45	11332.41	148.74	6235.99	5936.27	5070.00	0.85	6733.19	10545.80	15870.00	8211.04	20319.29	12357.41	12339.45	0.41	121.42	5821.25	8289.70	415.24	5563.06	5592.74	4300.57	0.76
137	P00746	CFAD_HUMAN	Complement factor D	secreted	x					5051.96	3345.34	3748.74	7210.63	3663.73	2612.37	4272.13	0.38	11010.72	9781.52	17267.72	20378.12	13303.32	15045.46	14464.48	0.27	11071.81	8951.78	17364.15	22458.33	15113.47	14797.86	14959.57	0.32	10229.78	8687.84	12799.99	16417.83	8733.69	10925.13	11299.04	0.26
138	Q9BX84	TRPM6_HUMAN	Transient receptor potential cation channel subfamily M member 6	leakage	x					2126.78	3643.18	5187.82	5344.88	5759.17	3343.99	4234.30	0.33	23669.00	25731.00	22947.13	33499.82	29232.45	25436.12	26752.58	0.15	53.03	39.89	48.81	58.35	261.48	53.07	85.77	1.01	1349.40	1212.33	1106.43	1688.00	1545.95	976.41	1313.09	0.21
139	P01763	HV348_HUMAN	Immunoglobulin heavy variable 3-48	secreted	x					6403.57	5250.67	3384.63	3690.89	3347.36	3072.64	4191.63	0.32	577.77	1005.69	687.02	874.07	900.96	717.56	793.84	0.20	1399.36	2921.61	4411.96	5364.35	5279.67	4882.26	4043.20	0.39	4035.16	5055.58	5353.79	7101.15	4809.69	6736.87	5515.37	0.21
140	P05154	IPSP_HUMAN	Plasma serine protease inhibitor	secreted	x					4930.75	5295.83	2599.01	4160.35	3977.54	3983.10	4157.77	0.23	9143.37	6496.62	6911.79	7392.11	6598.80	7136.05	7279.79	0.13	13663.66	9564.12	9665.34	10627.29	10804.83	10914.54	10873.30	0.14	12926.38	12915.23	11064.31	11433.79	13642.02	10825.55	12134.55	0.10
141	P18428	LBP_HUMAN	Lipopolysaccharide-binding protein	secreted	x					3853.93	4767.49	2814.00	1978.09	6107.34	4825.96	4057.80	0.37	14933.17	13035.17	17131.59	8488.02	14378.73	18682.90	14441.59	0.25	8635.79	10403.59	12408.98	8235.41	12396.37	16292.67	11395.47	0.26	4442.53	4268.55	5031.44	1757.08	5326.95	7392.65	4703.20	0.39
142	P04070	PROC_HUMAN	Vitamin K-dependent protein C	secreted	x					4676.92	3869.05	2596.50	3279.77	4416.02	3091.34	3654.93	0.22	4397.24	4435.41	3685.68	4132.29	4209.00	3375.56	4039.20	0.10	29823.45	21649.97	43548.79	38439.55	38306.37	33093.63	34143.63	0.23	27288.81	14542.29	24215.22	19318.00	27075.81	16270.06	21451.70	0.26
143	P14151	LYAM1_HUMAN	L-selectin	leakage	x					4091.07	3865.04	728.14	4733.61	4114.07	4059.11	3598.51	0.40	1978.71	2550.31	2055.12	2029.85	3260.75	1894.52	2294.88	0.23	982.15	1817.84	1438.53	2064.13	3276.32	1977.87	1926.14	0.40	859.83	1109.55	721.91	1205.98	1637.36	1143.40	1113.00	0.28
144	Q12805	FBLN3_HUMAN	EGF-containing fibulin-like extracellular matrix protein 1	secreted	x					4661.05	3900.42	1141.79	2917.69	3735.66	3748.78	3350.90	0.36	2156.97	2929.36	2015.07	1333.29	2277.25	2326.31	2173.04	0.24	1071.94	1942.00	1832.06	3023.53	4475.07	3276.97	2603.60	0.47	1085.26	1054.69	827.30	1078.86	1644.05	1317.40	1167.93	0.24
145	P26927	HGFL_HUMAN	Hepatocyte growth factor-like protein	secreted	x					5651.48	4394.11	1075.35	3336.15	2628.70	2352.62	3239.73	0.50	3336.80	3304.71	3094.79	1907.02	1690.84	642.86	2329.50	0.47	19871.37	25932.82	24306.83	26209.51	28009.37	20519.92	24141.64	0.14	36746.27	43229.80	37491.32	64622.43	63632.01	61515.66	51206.25	0.26
146	Q96A65	EXOC4_HUMAN	Exocyst complex component 4	not specified	x					1878.20	3406.59	3426.14	4212.46	3924.82	2345.20	3198.90	0.28	5345.91	5064.55	4969.08	4614.28	5727.07	4751.49	5078.73	0.08	198.91	139.82	171.78	155.81	387.26	313.18	227.79	0.44	1618.80	2018.12	1581.05	1343.69	1316.53	1886.59	1627.46	0.17
147	Q2M2I5	K1C24_HUMAN	Keratin, type I cytoskeletal 24	not specified	x					3360.57	3727.83	2200.00	2947.21	3718.69	2956.24	3151.76	0.18	673.50	920.30	1167.98	1548.73	957.87	2192.71	1243.51	0.44	3615.75	4481.53	4594.70	4884.27	5052.34	5854.15	4747.12	0.16	3517.99	4084.07	3245.55	2934.34	3520.32	3022.62	3387.48	0.12
148	B9A064	IGLL5_HUMAN	Immunoglobulin lambda-like polypeptide 5	secreted	x					3423.83	3796.18	3159.62	2475.61	1756.18	3043.54	2942.49	0.25	5913.61	4246.46	11870.54	4736.46	5239.63	9108.37	6852.51	0.44	7202.17	7426.53	12163.88	9504.04	4771.49	10483.02	8591.85	0.31	7679.46	6279.01	12405.81	4560.38	4834.90	6203.12	6993.78	0.41
149	P08571	CD14_HUMAN	Monocyte differentiation antigen CD14	secreted	x					2402.41	3532.37	2135.09	2875.42	3646.37	2178.26	2794.99	0.24	3170.21	5263.72	5993.96	3730.58	4250.76	3424.76	4305.67	0.26	3687.17	4223.56	6308.47	6611.11	7179.96	5626.85	5606.19	0.25	2542.04	4633.03	4439.45	3289.17	4268.50	2598.98	3628.53	0.26
150	P61769	B2MG_HUMAN	Beta-2-microglobulin [Cleaved into: Beta-2-microglobulin form pI 5.3]	secreted	x					2511.50	2960.08	1957.24	3549.95	3379.13	1978.40	2722.72	0.25	2720.66	3892.47	3681.68	4244.48	4522.72	3267.73	3721.62	0.18	2250.65	4149.21	3586.82	5134.83	3883.71	3136.43	3690.28	0.26	2945.69	3223.55	3365.79	4297.02	3493.60	3426.14	3458.63	0.13
151	Q9P2E3	ZNFX1_HUMAN	NFX1-type zinc finger-containing protein 1	not specified	x					2421.59	2748.36	4116.98	2269.82	2810.90	1586.20	2658.98	0.32	34400.36	35731.36	26623.13	24165.40	33463.65	26754.38	30189.71	0.16	3331.72	6261.51	3880.19	4972.67	3674.64	4117.01	4372.96	0.25	9654.05	9589.51	12006.60	7933.56	10276.26	6421.54	9313.59	0.21
152	Q9UK55	ZPI_HUMAN	Protein Z-dependent protease inhibitor	secreted	x					2667.47	2273.45	1736.20	2494.97	3373.72	3243.07	2631.48	0.23	11174.32	9972.88	11105.09	10295.20	10607.30	11883.53	10839.72	0.06	11173.69	9317.68	10098.06	12547.67	12872.12	14162.71	11695.32	0.16	12385.18	13700.54	14076.55	13524.05	16225.35	16741.39	14442.18	0.12
153	P01615	KVD28_HUMAN	Immunoglobulin kappa variable 2D-28	secreted	x					2454.83	2304.87	2764.49	2335.40	2574.78	2104.93	2423.22	0.09	2004.08	2164.52	4173.21	3195.82	3128.41	3346.29	3002.06	0.27	5037.66	2937.28	3907.08	4658.27	3281.30	3335.08	3859.44	0.22	711.64	416.35	783.30	629.14	603.77	384.98	588.20	0.27
154	P55058	PLTP_HUMAN	Phospholipid transfer protein	secreted	x					3390.33	2702.92	755.95	2327.45	2815.67	1934.94	2321.21	0.39	7485.54	6030.19	5625.96	8759.45	8004.45	6749.80	7109.23	0.17	38272.20	35611.44	26432.64	30038.11	22464.50	37741.61	31760.08	0.20	5094.15	4512.25	4451.66	3678.83	4668.53	2642.99	4174.73	0.21
155	P19013	K2C4_HUMAN	Keratin, type II cytoskeletal 4	not specified	x					2899.46	2478.87	849.63	2249.90	2180.76	2469.58	2188.03	0.32	3831.80	3088.96	4011.25	3102.54	3530.12	3913.43	3579.68	0.11	203.51	419.48	617.00	561.46	306.25	514.31	437.00	0.36	70.14	68.64	85.80	109.81	124.43	111.32	95.02	0.25
156	P68871	HBB_HUMAN	Hemoglobin subunit beta	not specified	x					5129.35	2569.17	689.17	1354.74	1177.93	2170.87	2181.87	0.73	9331.08	7136.49	5379.19	6400.47	4715.03	5102.89	6344.19	0.27	5560.21	3402.19	17205.67	3087.15	4325.52	4345.76	6321.08	0.85	5999.91	5708.29	5537.70	8279.14	5926.24	13816.35	7544.61	0.43
157	P55056	APOC4_HUMAN	Apolipoprotein C-IV	secreted	x					4922.36	1789.22	1343.74	1828.02	1446.41	1683.65	2168.90	0.63	6339.28	2243.18	2379.48	2911.40	2332.21	3376.06	3263.60	0.48	10743.55	3035.15	3137.21	3315.89	5528.30	4046.92	4967.84	0.60	6473.67	3945.83	3311.88	3982.65	3820.24	4060.82	4265.85	0.26
158	Q9NZP8	C1RL_HUMAN	Complement C1r subcomponent-like protein	secreted	x					2045.98	2742.32	804.93	1791.38	2340.73	2247.79	1995.52	0.33	3393.42	5929.38	5540.03	3685.19	4859.96	5054.69	4743.78	0.21	2107.52	3107.45	4221.81	4805.32	6056.84	5764.73	4343.95	0.35	7003.11	7963.06	6961.37	5281.10	10055.15	5742.18	7167.66	0.24
159	O95782	AP2A1_HUMAN	AP-2 complex subunit alpha-1	leakage	x					1025.44	1726.25	2198.22	2400.50	2348.98	1466.94	1861.06	0.30	8961.68	9637.57	11769.88	15783.79	12937.27	14542.65	12272.14	0.22	15246.29	11491.55	10668.84	14503.80	11554.01	12029.05	12582.26	0.15	688.60	725.34	645.82	578.07	984.90	905.31	754.67	0.21
160	Q08380	LG3BP_HUMAN	Galectin-3-binding protein	secreted	x					1770.54	1708.30	647.99	2086.50	1791.42	2904.31	1818.18	0.40	3024.57	2876.62	2548.20	3351.40	3175.49	4735.88	3285.36	0.23	3069.52	3474.42	3179.99	6472.50	5721.30	6842.48	4793.37	0.36	2633.64	1974.74	1682.25	3757.94	2650.10	4551.81	2875.08	0.38
161	Q07021	C1QBP_HUMAN	Complement component 1 Q subcomponent-binding protein, mitochondrial	secreted	x					2122.03	1736.13	587.73	2508.57	1157.32	2373.88	1747.61	0.43	76687.00	77557.22	82343.42	62973.88	97508.35	58888.57	75993.07	0.18	137.83	385.13	738.95	587.36	861.44	992.96	617.28	0.51	4770.56	4742.23	3348.39	7995.87	4257.21	8259.29	5562.26	0.37
162	P02533	K1C14_HUMAN	Keratin, type I cytoskeletal 14	leakage	x					466.20	338.53	170.92	895.41	333.65	7771.99	1662.78	1.81	5872.07	6407.73	4023.12	8614.92	11865.98	8053.86	7472.95	0.36	7064.42	8753.36	6863.70	5743.75	7147.93	7608.29	7196.91	0.14	741.30	1008.87	810.29	1767.61	1477.50	674.74	1080.05	0.41
163	P03951	FA11_HUMAN	Coagulation factor XI	secreted	x					2800.78	2062.88	452.74	1686.88	1249.80	1591.50	1640.76	0.48	25866.85	15656.61	13834.68	18438.44	16469.16	16403.85	17778.27	0.24	28860.62	18230.20	12593.80	19372.33	19599.80	16956.84	19268.93	0.28	1791.05	1416.88	1005.15	1403.30	1278.29	1195.89	1348.43	0.20
164	Q9P281	BAHC1_HUMAN	BAH and coiled-coil domain-containing protein 1	not specified	x					1603.25	1917.44	1376.95	1334.58	2072.73	1197.96	1583.82	0.22	692.36	452.00	1426.45	679.42	526.60	922.62	783.24	0.45	2663.01	1637.90	2146.68	1225.90	4018.20	1290.62	2163.72	0.49	5836.89	3628.96	4667.92	9346.88	2243.06	8836.45	5760.03	0.49
165	P09172	DOPO_HUMAN	Dopamine beta-hydroxylase	secreted	x					538.06	2498.24	1835.19	2248.48	786.96	1234.77	1523.62	0.52	3727.57	8164.02	6295.90	7652.83	2804.83	4278.08	5487.21	0.40	1408.44	6945.81	8637.41	9245.88	4607.13	6607.05	6241.95	0.46	2409.39	6904.44	7284.71	5825.14	3920.64	4832.28	5196.10	0.36
166	Q15582	BGH3_HUMAN	Transforming growth factor-beta-induced protein ig-h3	secreted	x					1824.01	1102.53	557.31	1433.65	1426.94	1398.75	1290.53	0.33	138709.75	133902.63	141492.83	137517.55	196894.17	126860.34	145896.21	0.17	3977.84	3178.34	5182.59	6254.81	9378.33	6041.31	5668.87	0.38	2144.70	1580.90	1888.66	2395.13	2131.79	2355.18	2082.73	0.15
167	P02775	CXCL7_HUMAN	Platelet basic protein	secreted	x					3098.39	1366.70	195.14	973.37	999.06	1066.45	1283.19	0.76	4124.58	9544.20	2474.47	4041.04	4859.61	2407.66	4575.26	0.57	1087.15	879.05	1118.97	824.38	3268.14	1408.68	1431.06	0.65	55817.20	66327.91	42268.11	58344.48	72303.29	61258.13	59386.52	0.17
168	P11226	MBL2_HUMAN	Mannose-binding protein C	secreted	x					1268.73	2252.25	666.76	1023.51	1334.78	951.44	1249.58	0.44	0.00	750.91	145.37	0.54	110.91	8.73	169.41	1.72	10100.17	15577.96	14315.25	16535.95	13859.21	12263.55	13775.35	0.17	113.74	1378.24	403.51	124.03	378.47	116.13	419.02	1.17
169	P17936	IBP3_HUMAN	Insulin-like growth factor-binding protein 3	secreted	x					1831.00	1337.09	97.10	1230.39	1212.99	1481.91	1198.42	0.49	2693.86	2563.61	1366.34	3317.38	2450.18	2298.58	2448.32	0.26	9121.02	8320.72	7011.61	12544.01	14041.80	10563.88	10267.17	0.26	8994.58	8479.72	7075.97	9276.72	12844.79	8281.05	9158.80	0.21
170	P01344	IGF2_HUMAN	Insulin-like growth factor II	secreted	x					1029.06	1038.75	657.03	1446.60	1194.92	1113.40	1079.96	0.24	9302.50	6412.55	8516.74	5798.82	5549.67	1424.18	6167.41	0.45	5428.34	3280.37	2974.91	3063.80	2805.11	1770.99	3220.59	0.37	2404.61	1787.59	3350.73	1491.17	801.72	2384.11	2036.65	0.43
171	P02741	CRP_HUMAN	C-reactive protein [Cleaved into: C-reactive protein	secreted	x					474.81	955.49	309.67	1076.89	817.84	2480.50	1019.20	0.76	2276.92	3379.61	3543.62	4117.04	3068.75	6195.77	3763.62	0.36	422.55	872.31	1033.83	928.32	1675.06	3232.05	1360.68	0.74	1.50	1.50	1.50	191.54	5.60	782.97	164.10	1.90
172	P14136	GFAP_HUMAN	Glial fibrillary acidic protein	leakage	x					861.47	1219.33	580.48	785.35	861.07	1235.46	923.86	0.28	5474.35	7387.85	9572.05	8575.95	9518.67	9560.32	8348.20	0.20	98953.45	65026.56	56423.07	55911.84	58367.48	53239.56	64653.66	0.27	9233.46	7791.40	6489.57	6924.68	13196.67	5158.28	8132.34	0.35
173	Q969I3	GLYL1_HUMAN	Glycine N-acyltransferase-like protein 1	not specified	x					1249.44	1146.71	228.30	815.51	410.90	996.12	807.83	0.51	997.37	1914.43	1333.51	1395.46	1302.27	2390.73	1555.63	0.32	10406.06	5637.74	5864.08	10127.55	7439.16	10496.79	8328.56	0.28	10332.88	12154.81	11101.05	14714.20	14405.37	12664.64	12562.16	0.14
174	P01764	HV323_HUMAN	Immunoglobulin heavy variable 3-23	secreted	x					962.69	701.81	628.27	638.40	671.61	998.56	766.89	0.22	7026.81	7640.63	6836.35	4524.04	5954.12	6048.29	6338.37	0.17	7914.89	7669.15	6292.85	7457.33	11970.71	9892.99	8532.99	0.24	1991.19	939.95	1552.90	1258.10	1019.02	1160.85	1320.33	0.30
175	P54108	CRIS3_HUMAN	Cysteine-rich secretory protein 3	secreted	x					882.74	951.77	313.97	578.88	912.29	889.09	754.79	0.34	1697.59	2417.73	2209.99	1521.00	2129.37	2165.14	2023.47	0.17	495.92	751.40	731.11	685.89	907.50	858.51	738.39	0.20	15273.31	30221.38	31514.70	46490.49	33196.11	46554.49	33875.08	0.35
176	P22891	PROZ_HUMAN	Vitamin K-dependent protein Z	secreted	x					1267.12	354.79	50.74	565.57	655.40	1342.96	706.10	0.72	1923.95	1592.45	3174.06	2003.20	2085.87	3124.52	2317.34	0.29	63055.94	53718.10	58111.64	43122.52	43198.10	41461.93	50444.71	0.18	6132.65	6062.67	7168.76	12922.73	7212.58	15470.63	9161.67	0.44
177	Q9UHG3	PCYOX_HUMAN	Prenylcysteine oxidase 1	leakage	x					729.98	1034.15	211.20	519.94	695.47	739.10	654.97	0.42	2020.37	2430.26	2252.16	1625.92	1963.27	2464.93	2126.15	0.15	3187.30	5848.30	4354.44	5448.72	6112.96	6907.57	5309.88	0.25	7663.92	6536.23	7179.44	8481.59	5659.02	8825.91	7391.02	0.16
178	P13646	K1C13_HUMAN	Keratin, type I cytoskeletal 13	not specified	x					711.87	862.29	121.93	627.12	295.69	1236.49	642.57	0.62	683.18	768.90	594.76	966.05	2792.84	776.41	1097.02	0.77	6173.20	23818.11	17271.86	4210.10	7390.99	6145.68	10834.99	0.73	3202.98	2822.17	3254.68	2595.60	4845.16	3499.23	3369.97	0.23
179	P01593	KVD33_HUMAN	Immunoglobulin kappa variable 1D-33	secreted	x					726.62	618.72	522.61	614.13	585.23	741.00	634.72	0.13	24735.71	31169.21	20147.54	29630.39	23250.70	38075.83	27834.90	0.23	1458.55	693.52	1930.26	1327.49	1447.91	1278.63	1356.06	0.29	935.53	712.25	1880.73	954.64	761.74	989.49	1039.06	0.41
180	P60709	ACTB_HUMAN	Actin, cytoplasmic 1	leakage	x					320.19	296.20	1.10	200.38	288.35	1965.15	511.89	1.41	9270.21	6665.40	2951.91	3097.91	4614.67	2724.66	4887.46	0.53	1813.88	510.21	4574.84	1969.03	4209.52	4841.01	2986.41	0.60	766.18	607.69	468.83	600.73	624.61	773.32	640.23	0.18
181	Q9BXR6	FHR5_HUMAN	Complement factor H-related protein 5	secreted	x					171.62	658.55	325.53	273.31	417.64	852.40	449.84	0.57	1771.48	1325.19	1228.46	942.36	1619.54	1101.75	1331.46	0.24	132.56	24.51	152.08	353.51	420.78	405.07	248.09	0.67	98.76	295.29	375.47	132.25	113.11	243.39	209.71	0.54
182	P49588	SYAC_HUMAN	Alanine--tRNA ligase, cytoplasmic	leakage	x					610.28	597.15	142.14	468.31	461.97	395.89	445.96	0.38	10757.53	8831.03	10401.40	9176.14	8497.78	9067.86	9455.29	0.10	7666.04	10049.37	6242.95	5831.48	7737.18	7465.06	7498.68	0.20	3589.70	3605.36	2939.44	4924.75	3062.12	6116.04	4039.57	0.31
183	Q9UIF8	BAZ2B_HUMAN	Bromodomain adjacent to zinc finger domain protein 2B	leakage	x					305.35	467.48	178.82	524.25	633.42	460.81	428.35	0.38	2513.26	3478.57	3718.93	3982.12	3020.51	3977.92	3448.55	0.17	91557.77	40810.48	53047.70	69542.20	47040.71	47255.70	58209.09	0.33	147.58	270.32	249.82	172.83	278.11	154.84	212.25	0.28
184	P06312	KV401_HUMAN	Immunoglobulin kappa variable 4-1	secreted	x					484.84	233.93	326.71	448.02	162.60	352.42	334.75	0.37	387.19	334.16	771.41	1163.15	585.90	911.50	692.22	0.46	1489.29	1594.21	1889.22	2467.38	726.66	1773.73	1656.75	0.34	1255.38	540.35	1305.92	1152.30	471.03	894.43	936.57	0.39
185	P01700	LV147_HUMAN	Immunoglobulin lambda variable 1-47	secreted	x					289.38	365.26	412.82	280.72	408.11	248.83	334.19	0.21	3722.02	3953.92	8671.67	5129.39	7293.16	8226.64	6166.13	0.35	432.77	490.02	630.09	473.25	1168.39	350.92	590.91	0.50	493.69	397.26	587.71	389.87	512.28	311.44	448.71	0.22
186	P12081	SYHC_HUMAN	Histidine--tRNA ligase, cytoplasmic	leakage	x					31.81	23.92	0.10	4.87	39.68	32.41	22.13	0.73	10403.52	6432.20	6468.75	9940.80	8592.43	6583.24	8070.16	0.23	16083.97	15631.91	11858.53	9128.49	11786.42	10402.59	12481.99	0.22	7848.45	8114.42	9527.41	7870.06	9084.76	9803.34	8708.08	0.10
187	P04406	G3P_HUMAN	Glyceraldehyde-3-phosphate dehydrogenase	leakage						178414.06	200998.84	171045.39	205295.69	282789.47	213518.42	208676.98	0.19	149.70	38.78	6.53	0.00	1969.91	0.00	360.82	2.19																
188	O75410	TACC1_HUMAN	Transforming acidic coiled-coil-containing protein 1	leakage		x				49174.24	65076.92	109983.84	54229.04	65833.02	39034.82	63888.65	0.39																								
189	Q8N9V7	TOPZ1_HUMAN	Testis- and ovary-specific PAZ domain-containing protein 1	leakage						40225.17	63750.82	67343.43	59060.61	71011.91	52945.53	59056.25	0.19	14955.83	11635.45	19333.68	13642.54	10507.82	15207.02	14213.72	0.22	16723.39	20298.31	12138.41	14792.21	12923.65	12529.61	14900.93	0.21								
190	Q14244	MAP7_HUMAN	Ensconsin	leakage						48468.50	45253.19	31528.82	34622.29	64192.41	34468.82	43089.01	0.29	630.10	888.73	1465.66	958.63	943.85	1660.28	1091.21	0.36	33474.05	24942.57	28903.40	37474.20	23653.88	28503.01	29491.85	0.18								
191	P29508	SPB3_HUMAN	Serpin B3	secreted						35650.87	42598.15	48009.99	36783.00	58354.86	31016.37	42068.87	0.24	7316.50	10624.62	9684.44	11080.11	14286.01	10057.70	10508.23	0.22									12213.17	18038.40	14934.12	18700.20	15013.33	14947.31	15641.09	0.15
192	Q9P2D6	F135A_HUMAN	Protein FAM135A	not specified		x				47424.57	51139.66	9049.49	33083.89	57915.08	46010.11	40770.47	0.43																								
193	Q99683	M3K5_HUMAN	Mitogen-activated protein kinase kinase kinase 5	leakage		x				37557.78	44892.96	34789.61	36855.85	52332.79	37776.02	40700.83	0.16																								
194	Q15751	HERC1_HUMAN	Probable E3 ubiquitin-protein ligase HERC1	leakage						47848.64	48967.19	27399.96	36202.87	39778.46	40468.77	40110.98	0.20	58676.87	74441.11	73299.88	66869.23	60506.39	79897.04	68948.42	0.12																
195	P49747	COMP_HUMAN	Cartilage oligomeric matrix protein	secreted						49884.88	46653.04	16167.50	44568.68	39144.90	29373.14	37632.02	0.34									67060.32	68123.32	37962.87	68439.40	55888.39	55564.96	58839.88	0.20								
196	Q8TCU6	PREX1_HUMAN	Phosphatidylinositol 3,4,5-trisphosphate-dependent Rac exchanger 1 protein	leakage						28757.85	31419.45	21030.16	26762.66	36495.93	32925.75	29565.30	0.18	2049.17	2066.22	2241.33	1960.34	1843.51	2209.54	2061.68	0.07																
197	Q9BYG8	GSDMC_HUMAN	Gasdermin-C	leakage						29162.14	31746.45	19120.90	26162.30	30964.89	26829.71	27331.07	0.17									2763.48	1702.73	1360.53	2086.75	1169.04	1937.59	1836.69	0.31								
198	Q9Y6X9	MORC2_HUMAN	MORC family CW-type zinc finger protein 2	leakage						29871.92	25435.73	20320.72	24536.41	28002.91	25875.34	25673.84	0.13	39329.33	32488.72	33396.26	25850.53	27088.00	27556.72	30951.59	0.17	28101.07	26106.63	26724.73	34626.09	33567.75	30186.19	29885.41	0.12								
199	Q9P0W8	SPAT7_HUMAN	Spermatogenesis-associated protein 7	leakage		x				21639.73	32866.08	28811.45	17938.46	32731.87	19728.95	25619.42	0.26																								
200	Q9BYW2	SETD2_HUMAN	Histone-lysine N-methyltransferase SETD2	leakage		x				22027.35	26582.71	21030.25	22560.70	30931.18	20782.97	23985.86	0.17																								
201	Q15573	TAF1A_HUMAN	TATA box-binding protein-associated factor RNA polymerase I subunit A	leakage		x				14670.98	20078.20	22198.96	21065.31	27358.77	18242.93	20602.52	0.21																								
202	Q8IWC1	MA7D3_HUMAN	MAP7 domain-containing protein 3	leakage						24354.86	25055.77	12029.47	21859.04	11159.07	28784.64	20540.47	0.35	35758.40	47130.36	53969.06	38009.29	49197.19	44250.18	44719.08	0.15	30393.12	20861.18	14505.68	17687.19	32827.71	38672.55	25824.57	0.37								
203	Q16787	LAMA3_HUMAN	Laminin subunit alpha-3	secreted						18323.49	21631.60	18180.16	19931.33	23197.25	13774.88	19173.12	0.17	8512.02	8505.39	9258.69	9836.30	8671.91	9637.57	9070.31	0.06																
204	P15813	CD1D_HUMAN	Antigen-presenting glycoprotein CD1d	leakage		x				13394.73	17279.88	13397.98	14071.09	20923.29	15274.64	15723.60	0.19																								
205	Q86UK0	ABCAC_HUMAN	ATP-binding cassette sub-family A member 12	leakage						13075.92	14066.23	18238.38	11576.73	17426.47	7271.71	13609.24	0.30	31773.44	42092.59	88313.63	59471.67	68333.83	86419.58	62734.12	0.37																

Supplementary material Overview on all sample collection methods relatively quantified proteins after depletion

										EDTA plasma samples								heparin plasma samples								citrate plasma samples								serum samples							
Nr	Accession	Entry name	Protein names	secreted/ leakage	core set	unique EDTA	unique heparin	unique citrate	unique serum	EDTA_1	EDTA_2	EDTA_3	EDTA_4	EDTA_5	EDTA_6	Mean abundance	CV	heparin_1	heparin_2	heparin_3	heparin_4	heparin_5	heparin_6	Mean abundance	CV	citrate_1	citrate_2	citrate_3	citrate_4	citrate_5	citrate_6	Mean abundance	CV	serum_1	serum_2	serum_3	serum_4	serum_5	serum_6	Mean abundance	CV
206	Q9Y2M0	FAN1_HUMAN	Fanconi-associated nuclease 1	leakage						14243.16	23268.32	9090.45	8380.76	12048.53	6870.45	12316.95	0.49																	61732.93	90269.02	65925.24	70179.53	72031.19	65704.05	70973.66	0.14
207	P00488	F13A_HUMAN	Coagulation factor XIII A chain	secreted						13195.21	11742.48	8615.96	12293.81	14314.07	9696.73	11643.04	0.18	18770.27	14948.60	19317.55	22492.86	16579.43	19840.48	18658.20	0.14	7122.20	4206.52	6311.77	6709.31	6016.45	5690.13	6009.40	0.17								
208	O14950	ML12B_HUMAN	Myosin regulatory light chain 12B	not specified		x				10508.12	9973.55	11783.63	13290.69	8753.26	9291.25	10600.08	0.16																								
209	Q15323	K1H1_HUMAN	Keratin, type I cuticular Ha1	not specified						10390.97	10122.12	6678.11	7645.76	12738.01	7698.31	9212.21	0.25									0.01	0.01	15113.37	0.01	0.14	0.08	2518.94	2.45								
210	P08729	K2C7_HUMAN	Keratin, type II cytoskeletal 7	leakage						6643.91	11999.01	14103.42	6169.77	6382.61	6566.02	8644.12	0.40									4283.71	12627.40	5484.38	7132.89	3895.67	6387.50	6635.26	0.48	1290.13	1463.75	1237.83	1166.18	1403.56	997.15	1259.77	0.13
211	O43719	HTSF1_HUMAN	HIV Tat-specific factor 1	leakage						7245.31	8942.01	7399.48	8753.86	11254.16	6856.80	8408.60	0.19	376.90	417.73	594.40	370.85	492.76	613.62	477.71	0.22																
212	Q5TBE3	CI153_HUMAN	Uncharacterized protein C9orf153	not specified		x				6183.02	7638.52	8439.16	7543.46	11013.53	7127.78	7990.91	0.21																								
213	Q12802	AKP13_HUMAN	A-kinase anchor protein 13	leakage						6994.68	8978.82	4214.41	8845.45	10257.20	7511.47	7800.34	0.27	4077.67	4235.99	5348.15	2937.44	4786.47	4305.15	4281.81	0.19	14581.40	10985.93	13630.65	11650.18	14905.96	9474.83	12538.16	0.17								
214	Q8N4P6	LRC71_HUMAN	Leucine-rich repeat-containing protein 71	not specified		x				10463.23	8449.77	5703.26	7122.59	6757.66	6869.22	7560.95	0.22																								
215	Q8IWZ5	TRI42_HUMAN	Tripartite motif-containing protein 42	not specified						7662.24	8078.89	6429.45	10006.48	6491.45	5999.14	7444.61	0.20																	5137.72	5836.99	5769.43	7672.98	5409.56	4509.54	5722.70	0.19
216	Q14289	FAK2_HUMAN	Protein-tyrosine kinase 2-beta	leakage						5883.85	4959.77	14793.36	5276.93	7626.60	3360.04	6983.43	0.58									3263.37	5067.88	4986.43	5621.33	4133.58	5484.48	4759.51	0.19	399.74	540.87	486.22	486.26	455.20	510.09	479.73	0.10
217	P02538	K2C6A_HUMAN	Keratin, type II cytoskeletal 6A	not specified						8122.72	6593.36	4818.93	5628.90	5404.04	6941.61	6251.59	0.19	0.00	390.65	28.71	12.81	6696.90	53.38	1197.08	2.25									47.32	20.94	34.25	17.00	483.33	70.10	112.16	1.63
218	Q9BRR9	RHG09_HUMAN	Rho GTPase-activating protein 9	not specified		x				3711.66	5166.37	4631.88	7074.73	11585.27	4864.02	6172.32	0.47																								
219	P05783	K1C18_HUMAN	Keratin, type I cytoskeletal 18	leakage						7814.53	7091.10	3012.45	6547.89	5968.54	6129.13	6093.94	0.27									0.01	0.01	10.57	50.76	50.35	7.87	19.93	1.21								
220	Q3KQU3	MA7D1_HUMAN	MAP7 domain-containing protein 1	leakage						6798.79	6525.27	4280.39	6979.31	4981.88	6302.38	5978.00	0.18	964.37	1197.40	1789.86	829.26	631.63	1810.55	1203.85	0.41	57902.11	50691.21	25619.45	21237.49	37937.91	43301.20	39448.23	0.36								
221	P06702	S10A9_HUMAN	Protein S100-A9	secreted		x				2987.84	3691.38	6077.79	5006.49	4928.26	11813.18	5750.82	0.55																								
222	Q8WZ64	ARAP2_HUMAN	Arf-GAP with Rho-GAP domain, ANK repeat and PH domain-containing protein 2	leakage						4901.51	6021.47	4354.50	5715.94	6090.86	5013.87	5349.69	0.13	3611.37	3140.99	7137.21	8536.14	3363.69	6325.53	5352.49	0.43																
223	Q86VH2	KIF27_HUMAN	Kinesin-like protein KIF27	leakage						4698.81	4314.72	4255.93	3858.23	7596.55	3819.71	4757.33	0.30	40553.46	42520.81	38860.60	31113.92	49477.14	34069.65	39432.60	0.16	30971.96	17414.14	20042.65	24713.26	29954.82	25953.85	24841.78	0.22								
224	Q12929	EPS8_HUMAN	Epidermal growth factor receptor kinase substrate 8	leakage						3927.08	4300.83	2046.15	5973.85	3879.45	7460.99	4598.06	0.41																	977.85	1441.35	999.43	1170.10	750.23	1095.13	1072.35	0.21
225	P0CG06	LAC3_HUMAN	Ig lambda-3 chain C regions	not specified						3890.32	5637.60	7168.33	3622.65	3199.59	2731.64	4375.02	0.39	22807.67	15123.66	22783.71	15787.63	11446.47	14672.04	17103.53	0.27	25969.32	23995.66	26769.60	22743.40	9911.17	17793.14	21197.05	0.30	59.47	39.51	57.46	66.00	95.84	87.91	67.70	0.31
226	P08779	K1C16_HUMAN	Keratin, type I cytoskeletal 16	not specified						3351.22	3960.96	2644.22	3246.18	3954.07	8722.45	4313.18	0.51	1473.28	4535.58	1162.91	1272.45	32339.24	1232.91	7002.73	1.78	723.29	435.09	557.40	1191.62	1237.35	1354.22	916.50	0.43								
227	Q9Y4A0	JERKL_HUMAN	Jerky protein homolog-like	leakage		x				4983.09	4430.73	3304.30	4131.30	3598.12	3976.11	4070.61	0.15																								
228	P50454	SERPH_HUMAN	Serpin H1	leakage		x				3583.10	4501.81	3363.32	3800.85	4957.99	3363.69	3928.46	0.17																								
229	Q6ZN16	M3K15_HUMAN	Mitogen-activated protein kinase kinase kinase 15	not specified						3962.18	3476.81	2681.74	3005.66	7029.41	2686.70	3807.08	0.43	6405.49	6671.74	8680.32	5498.34	4956.83	8271.42	6747.36	0.22	4126.40	2724.71	2792.11	3998.14	2100.60	3244.37	3164.39	0.25								
230	O60443	DFNA5_HUMAN	Non-syndromic hearing impairment protein 5	leakage		x				3414.44	4399.90	2867.77	4125.34	5040.06	2762.26	3768.30	0.24																								
231	P06213	INSR_HUMAN	Insulin receptor	leakage		x				4374.83	3735.01	2744.13	4798.05	2925.51	3899.22	3746.12	0.21																								
232	P10916	MLRV_HUMAN	Myosin regulatory light chain 2, ventricular/cardiac muscle isoform	leakage						4435.58	2252.95	3011.89	5495.31	4034.85	2423.72	3609.05	0.35	4002.26	3852.15	3760.48	4616.83	3210.64	2581.11	3670.58	0.19																
233	Q86Y82	STX12_HUMAN	Syntaxin-12	leakage		x				2185.67	3048.33	921.16	6898.49	4167.14	3563.62	3464.07	0.59																								
234	Q92954	PRG4_HUMAN	Proteoglycan 4	secreted		x				2883.76	3634.98	2337.93	4016.26	3599.57	4053.29	3420.97	0.20																								
235	Q8N335	GPD1L_HUMAN	Glycerol-3-phosphate dehydrogenase 1-like protein	leakage		x				2956.18	2621.56	4718.96	2883.10	5217.53	1968.10	3394.24	0.38																								
236	Q8IXS0	F217A_HUMAN	Protein FAM217A	not specified		x				3970.24	3922.53	1348.96	3212.30	3576.54	3890.47	3320.17	0.30																								
237	Q3MIT2	PUS10_HUMAN	Putative tRNA pseudouridine synthase Pus10	not specified		x				3486.86	3633.73	2023.13	3469.91	3175.14	3910.55	3283.22	0.20																								
238	P57076	CU059_HUMAN	UPF0769 protein C21orf59	leakage		x				3508.33	4014.61	4242.48	3004.21	2646.58	2069.87	3247.68	0.26																								
239	P13647	K2C5_HUMAN	Keratin, type II cytoskeletal 5	not specified						1916.04	1976.91	1593.46	2414.04	1621.94	8726.06	3041.41	0.92	83.94	229.10	243.57	205.47	1222.15	252.90	372.86	1.13	9916.54	11498.74	11630.36	9517.42	9882.94	10577.15	10503.86	0.08								
240	Q8NF67	A2012_HUMAN	Putative ankyrin repeat domain-containing protein 20A12 pseudogene	not specified		x				3949.47	3725.12	1561.71	3320.02	318.99	4531.29	2901.10	0.56																								
241	P07316	CRGB_HUMAN	Gamma-crystallin B	not specified						3040.68	3035.30	2743.77	2289.68	3774.16	1920.68	2800.71	0.23	1192.56	1075.85	1357.86	1344.82	883.94	2174.40	1338.24	0.33									4869.15	7302.27	7338.08	6257.95	9527.42	6686.82	6996.95	0.22
242	Q7Z406	MYH14_HUMAN	Myosin-14	not specified		x				3098.02	2701.14	2104.81	3150.50	2559.53	2820.44	2739.07	0.14																								
243	Q5TZA2	CROCC_HUMAN	Rootletin	leakage						3462.40	3740.89	1270.60	1849.54	2864.90	2437.31	2604.27	0.36	5209.64	6104.03	8198.88	5107.10	6837.44	6955.32	6402.07	0.18	8801.49	2517.65	5584.35	6880.08	10979.10	7822.59	7097.54	0.41								
244	O00391	QSOX1_HUMAN	Sulfhydryl oxidase 1	secreted						2179.11	2069.91	1177.66	1957.69	6134.28	1921.16	2573.30	0.69	2110.28	2616.26	3111.49	1619.61	1893.58	2743.06	2349.05	0.24	3296.04	3403.13	8537.66	6022.12	2698.78	5262.19	4869.99	0.45								
245	Q96EI5	TCAL4_HUMAN	Transcription elongation factor A protein-like 4	leakage						1670.45	3067.02	2633.15	2385.18	3310.38	1489.25	2425.90	0.30	6387.66	7057.11	5187.69	6731.57	6864.42	7021.01	6541.58	0.11																
246	Q6W2J9	BCOR_HUMAN	BCL-6 corepressor	leakage		x				2415.88	2982.58	1670.47	2003.58	2424.46	2160.41	2276.23	0.20																								
247	P05109	S10A8_HUMAN	Protein S100-A8	secreted						2415.91	2193.06	1536.06	3124.96	377.30	3263.56	2151.81	0.50	2677.59	2137.23	1799.37	3608.71	1547.03	3059.88	2471.64	0.32																
248	P01034	CYTC_HUMAN	Cystatin-C	secreted						2145.28	1825.50	1684.24	2463.42	2877.09	1850.86	2141.06	0.21	1097.99	1674.15	1844.06	1013.99	2035.09	1557.91	1537.20	0.26	1486.85	2214.26	1867.15	2737.36	1508.19	2274.68	2014.75	0.24								
249	H7BZ55	CRCC2_HUMAN	Putative ciliary rootlet coiled-coil protein 2	not specified		x				2427.46	2209.82	1061.85	1958.09	2231.37	2304.30	2032.15	0.25																								
250	P31151	S10A7_HUMAN	Protein S100-A7	secreted		x				1415.23	1232.61	708.84	1326.56	1251.12	6137.33	2011.95	1.01																								
251	Q8IV77	CNGA4_HUMAN	Cyclic nucleotide-gated cation channel alpha-4	leakage		x				2344.95	2226.07	713.49	2000.66	1922.12	2562.13	1961.57	0.33																								
252	Q13474	DRP2_HUMAN	Dystrophin-related protein 2	leakage						1754.77	2666.63	1297.22	2468.59	2131.18	1370.24	1948.10	0.29	10335.02	10006.35	6875.64	10116.61	9594.09	3704.63	8438.72	0.31																
253	Q14141	SEPT6_HUMAN	Septin-6	leakage		x				2535.62	2210.08	983.81	1687.17	1260.13	2121.24	1799.68	0.33																								
254	C9J302	CD051_HUMAN	Uncharacterized protein C4orf51	not specified		x				1489.25	2065.97	1840.40	1696.85	2254.38	1391.07	1789.65	0.19																								
255	Q86U86	PB1_HUMAN	Protein polybromo-1	leakage						1609.74	2097.20	521.52	2378.97	1557.57	1986.58	1691.93	0.38	4609.93	7182.25	8169.41	9076.04	8578.37	9539.42	7859.24	0.23																
256	Q7Z3Z0	K1C25_HUMAN	Keratin, type I cytoskeletal 25	leakage						1815.18	414.95	44.78	2372.36	262.68	4844.80	1625.79	1.13									1523.40	1273.63	1570.18	2679.16	2884.51	2143.46	2012.39	0.33	322.06	92.34	177.57	65.52	471.50	54.48	197.24	0.85
257	Q9C029	TRIM7_HUMAN	Tripartite motif-containing protein 7	not specified		x				1739.32	2131.79	937.38	1474.86	1768.00	1462.55	1585.65	0.25																								
258	Q92598	HS105_HUMAN	Heat shock protein 105 kDa	leakage						1368.31	1460.63	1665.37	1176.72	2151.97	1177.49	1500.08	0.25	207350.34	194182.00	146084.36	165976.47	170585.16	142492.00	171111.72	0.15	8083.35	5951.47	7759.03	7907.02	9216.40	8901.71	7969.83	0.14								
259	P07477	TRY1_HUMAN	Trypsin-1	secreted						1336.97	1963.70	340.24	1404.69	2128.10	1500.17	1445.64	0.43									43836.43	54133.50	42050.73	42829.50	39448.00	50039.96	45389.69	0.12								
260	P47929	LEG7_HUMAN	Galectin-7	secreted						242.23	132.45	24.20	134.82	288.79	7514.94	1389.57	2.16	0.00	0.51	12.88	0.00	987.20	12.83	168.90	2.37																
261	P27482	CALL3_HUMAN	Calmodulin-like protein 3	not specified		x				1318.28	1530.43	682.67	1282.74	1552.39	1309.24	1279.29	0.25																								
262	Q9NZT1	CALL5_HUMAN	Calmodulin-like protein 5	not specified						437.82	474.50	90.95	415.26	1412.48	4592.26	1237.21	1.38	8683.83	8178.79	10600.85	7385.14	14120.94	7459.54	9404.85	0.28																
263	Q6VMQ6	MCAF1_HUMAN	Activating transcription factor 7-interacting protein 1	leakage						881.73	1874.44	1063.11	764.14	899.67	649.09	1022.03	0.43									272.91	8945.73	1470.50	1017.85	1924.97	1215.92	2474.64	1.30	14541.72	19611.51	15471.87	15407.52	13663.11	11350.51	15007.71	0.18
264	Q14CN4	K2C72_HUMAN	Keratin, type II cytoskeletal 72	not specified		x				1522.80	1243.99	69.56	859.79	649.67	1227.56	928.90	0.56																								
265	Q02410	APBA1_HUMAN	Amyloid beta A4 precursor protein-binding family A member 1	leakage		x				1406.35	1172.74	357.73	1172.08	474.41	908.25	915.26	0.46																								
266	A0PJZ0	A20A5_HUMAN	Putative ankyrin repeat domain-containing protein 20A5	not specified		x				1030.51	1020.22	22.09	1186.89	861.12	1167.48	881.39	0.50																								
267	P22532	SPR2D_HUMAN	Small proline-rich protein 2D	leakage		x				182.28	211.27	20.77	242.03	316.38	4096.39	844.85	1.89																								
268	P04792	HSPB1_HUMAN	Heat shock protein beta-1	leakage		x				165.41	232.94	0.10	625.49	217.32	3702.30	823.93	1.73																								
269	Q9UBP9	GULP1_HUMAN	PTB domain-containing engulfment adapter protein 1	leakage		x				702.11	1117.62	157.71	646.15	783.57	1433.51	806.78	0.54																								
270	Q5TG30	RHG40_HUMAN	Rho GTPase-activating protein 40	not specified						910.01	958.39	123.50	1180.70	704.59	827.92	784.19	0.46	925.00	2023.10	1738.98	2195.82	2173.24	2673.35	1954.92	0.30	3206.60	2488.22	6527.10	9808.07	6479.28	8480.93	6165.03	0.47								
271	P05787	K2C8_HUMAN	Keratin, type II cytoskeletal 8	leakage						1352.82	912.30	312.94	657.95	287.01	991.53	752.42	0.55									92.64	80.64	223.01	339.05	225.22	213.98	195.76	0.49	8236.48	8466.29	8973.93	9945.35	8770.58	11556.04	9324.78	0.13
272	Q6ZU80	CE128_HUMAN	Centrosomal protein of 128 kDa	leakage						653.54	753.95	368.02	634.81	711.99	789.14	651.91	0.23																	794.65	882.85	728.63	1062.28	655.56	881.98	834.32	0.17
273	Q86Z20	CC125_HUMAN	Coiled-coil domain-containing protein 125	leakage						457.17	635.67	402.08	701.40	648.36	525.15	561.64	0.21									23.75	1429.43	113.56	54.79	109.71	103.82	305.84	1.80								
274	P28072	PSB6_HUMAN	Proteasome subunit beta type-6	leakage		x				137.46	319.07	1766.60	277.63	516.07	197.71	535.76	1.15																								
275	Q7RTS7	K2C74_HUMAN	Keratin, type II cytoskeletal 74	not specified						439.77	384.47	587.45	461.69	797.64	272.86	490.65	0.37	1453.75	1762.96	888.13	2210.09	1817.70	1111.93	1540.76	0.32																
276	P69905	HBA_HUMAN	Hemoglobin subunit alpha	not specified						1123.59	634.52	16.85	298.16	270.84	390.35	455.72	0.84	1993.36	1930.94	1092.96	496.28	804.00	823.18	1190.12	0.53									523.28	234.76	355.61	558.06	326.70	1886.40	647.47	0.96
277	Q8NHP7	EXD1_HUMAN	piRNA biogenesis protein EXD1	leakage		x				532.14	545.52	206.51	427.58	529.50	368.40	434.94	0.30																								
278	Q5T749	KPRP_HUMAN	Keratinocyte proline-rich protein	leakage		x				77.14	77.54	0.10	22.88	0.10	2413.64	431.90	2.25																								
279	P00915	CAH1_HUMAN	Carbonic anhydrase 1	leakage						1266.80	182.58	0.10	384.87	285.60	278.91	399.81	1.11	664.22	525.94	503.19	210.37	365.76	297.99	427.91	0.39									91.39	37.60	76.89	97.29	202.62	340.01	140.97	0.79
280	Q04695	K1C17_HUMAN	Keratin, type I cytoskeletal 17	leakage						22.73	19.51	0.10	1103.47	12.49	1212.35	395.11	1.50	129.61	349.53	144.37	99.83	33118.93	198.61	5673.48	2.37																
281	Q13588	GRAP_HUMAN	GRB2-related adapter protein	not specified		x				542.84	524.58	31.73	353.38	302.52	459.97	369.17	0.52																								
282	Q8TCY9	URGCP_HUMAN	Up-regulator of cell proliferation	leakage						225.52	360.28	16.22	692.54	218.64	691.41	367.43	0.75	412.36	495.59	540.69	448.74	399.09	465.89	460.39	0.11																
283	Q7Z3Y9	K1C26_HUMAN	Keratin, type I cytoskeletal 26	not specified		x				515.20	353.00	197.59	379.26	409.84	261.68	352.76	0.32																								
284	Q9UBB4	ATX10_HUMAN	Ataxin-10	leakage		x				571.64	492.81	170.03	373.94	181.11	146.50	322.67	0.57																								
285	P0DJI8	SAA1_HUMAN	Serum amyloid A-1 protein	secreted						219.66	327.10	0.10	871.66	217.15	275.94	318.61	0.92	2293.49	4329.76	3027.48	9511.07	4453.18	3755.69	4561.78	0.56									3076.19	7842.10	3802.11	19181.84	6124.52	5114.37	7523.52	0.79
286	P01619	KV320_HUMAN	Immunoglobulin kappa variable 3-20	secreted						128.08	695.60	383.68	153.43	88.09	217.15	277.67	0.83									5338.23	2967.54	3882.71	3215.41	1841.40	3109.58	3392.48	0.34								
287	Q08554	DSC1_HUMAN	Desmocollin-1	leakage		x				95.10	40.57	3.81	59.99	46.87	1383.52	271.65	2.01																								
288	Q9UPV7	PHF24_HUMAN	PHD finger protein 24	not specified		x				122.40	254.29	152.15	336.53	419.94	179.06	244.06	0.47																								
289	P13521	SCG2_HUMAN	Secretogranin-2	secreted		x				321.08	323.30	214.18	287.24	171.83	144.75	243.73	0.32																								
290	O75436	VP26A_HUMAN	Vacuolar protein sorting-associated protein 26A	leakage		x				217.53	229.30	25.47	380.19	318.30	170.08	223.48	0.55																								
291	Q9UHE5	NAT8_HUMAN	N-acetyltransferase 8	leakage		x				354.33	337.71	65.94	142.22	197.40	122.24	203.31	0.58																								
292	Q9BU64	CENPO_HUMAN	Centromere protein O	leakage		x				220.59	257.58	15.03	309.92	179.95	218.91	200.33	0.50																								
293	Q96A32	MLRS_HUMAN	Myosin regulatory light chain 2, skeletal muscle isoform	not specified		x				357.22	451.31	0.10	86.87	87.13	134.66	186.22	0.95																								
294	Q02325	PLGB_HUMAN	Plasminogen-like protein B	secreted						253.96	237.52	68.32	154.52	21.27	334.46	178.34	0.67	203.73	224.29	319.05	248.28	218.04	217.00	238.40	0.18	151.10	125.06	229.67	268.62	214.81	452.83	240.35	0.49								
295	Q9NQ66	PLCB1_HUMAN	1-phosphatidylinositol 4,5-bisphosphate phosphodiesterase beta-1	leakage		x				223.95	150.15	10.89	161.85	109.77	311.67	161.38	0.63																								
296	Q01469	FABP5_HUMAN	Fatty acid-binding protein, epidermal	leakage		x				123.59	56.95	0.10	34.37	23.00	489.49	121.25	1.53																								
297	P0CG47	UBB_HUMAN	Polyubiquitin-B [Cleaved into: Ubiquitin]	leakage		x				101.70	46.51	0.10	21.62	28.57	500.33	116.47	1.64																								
298	Q1L5Z9	LONF2_HUMAN	LON peptidase N-terminal domain and RING finger protein 2	not specified		x				236.11	136.70	0.10	55.52	60.83	198.86	114.69	0.80																								
299	P0C1S8	WEE2_HUMAN	Wee1-like protein kinase 2	leakage		x				281.85	18.68	3.82	122.11	17.04	202.03	107.59	1.07																								
300	Q8N7B9	EFCB3_HUMAN	EF-hand calcium-binding domain-containing protein 3	not specified		x				182.71	196.49	10.11	105.66	87.92	23.75	101.11	0.77																								
301	P32456	GBP2_HUMAN	Guanylate-binding protein 2	leakage		x				191.56	141.63	0.10	34.09	60.76	166.82	99.16	0.79																								
302	P41222	PTGDS_HUMAN	Prostaglandin-H2 D-isomerase	secreted		x				68.52	119.10	0.10	138.81	165.74	67.80	93.35	0.64																								
303	Q9ULV0	MYO5B_HUMAN	Unconventional myosin-Vb	leakage						244.04	96.46	0.10	49.07	23.34	138.47	91.92	0.98	244.32	321.17	368.90	323.62	443.95	270.68	328.77	0.22									57.06	13.45	86.48	44.04	28.80	54.31	47.36	0.53
304	Q02045	MYL5_HUMAN	Myosin light chain 5	not specified						112.81	154.57	30.04	81.27	99.40	69.83	91.32	0.46	15764.72	20422.56	35945.18	28184.19	27372.20	34465.61	27025.74	0.29	7677.77	12923.67	29986.90	36734.97	29558.71	34415.35	25216.23	0.48								
305	Q14676	MDC1_HUMAN	Mediator of DNA damage checkpoint protein 1	leakage		x				52.53	74.36	0.10	116.46	67.45	155.50	77.73	0.69																								

Supplementary material Overview on all sample collection methods relatively quantified proteins after depletion

										EDTA plasma samples								heparin plasma samples								citrate plasma samples								serum samples							
Nr	Accession	Entry name	Protein names	secreted/ leakage	core set	unique EDTA	unique heparin	unique citrate	unique serum	EDTA_1	EDTA_2	EDTA_3	EDTA_4	EDTA_5	EDTA_6	Mean abundance	CV	heparin_1	heparin_2	heparin_3	heparin_4	heparin_5	heparin_6	Mean abundance	CV	citrate_1	citrate_2	citrate_3	citrate_4	citrate_5	citrate_6	Mean abundance	CV	serum_1	serum_2	serum_3	serum_4	serum_5	serum_6	Mean abundance	CV
306	O95155	UBE4B_HUMAN	Ubiquitin conjugation factor E4 B	leakage						39.32	17.73	14.75	152.60	9.96	115.83	58.37	1.04									1406.40	974.82	797.27	1793.33	1990.71	1651.11	1435.60	0.33								
307	O95497	VNN1_HUMAN	Pantetheinase	leakage						73.38	34.51	0.10	40.88	0.10	186.01	55.83	1.24																	4070.76	4831.08	4354.18	4261.88	3539.69	4471.73	4254.88	0.10
308	Q8TF21	ANR24_HUMAN	Ankyrin repeat domain-containing protein 24	not specified						93.98	36.51	0.10	11.67	9.41	77.13	38.13	1.02									4325.70	3007.42	4161.95	4450.75	4723.19	3592.95	4043.66	0.16								
309	P48426	PI42A_HUMAN	Phosphatidylinositol 5-phosphate 4-kinase type-2 alpha	leakage						42.48	15.43	0.10	8.72	8.58	8.74	14.01	1.05									185.35	303.48	1215.69	2580.09	1701.76	1408.28	1232.44	0.73								
310	Q9NUI1	DECR2_HUMAN	Peroxisomal 2,4-dienoyl-CoA reductase	leakage		x				23.86	20.03	0.10	3.72	0.10	31.19	13.17	1.03																								
311	Q9HC77	CENPJ_HUMAN	Centromere protein J	leakage						5.39	0.10	0.10	16.01	0.10	29.30	8.50	1.40	11319.70	10493.22	9361.66	8733.66	9242.49	8462.01	9602.12	0.11	200185.94	146906.59	258658.86	385065.03	318014.31	371055.16	279980.98	0.34								
312	P04275	VWF_HUMAN	von Willebrand factor	secreted					x																									171296.30	202738.97	192598.20	166870.00	183833.36	187043.91	184063.46	0.07
313	O95602	RPA1_HUMAN	DNA-directed RNA polymerase I subunit RPA1	leakage					x																									118817.42	176926.23	184007.03	192231.88	145287.61	177692.09	165827.04	0.17
314	Q13439	GOGA4_HUMAN	Golgin subfamily A member 4	leakage														4201.34	3799.80	8640.93	5914.67	5676.81	6902.49	5856.01	0.30									142185.69	184730.91	150695.77	159634.38	190007.58	115869.38	157187.28	0.18
315	Q9ULI0	ATD2B_HUMAN	ATPase family AAA domain-containing protein 2B	leakage					x																									51935.42	64557.26	22305.82	53281.60	62255.47	57282.29	51936.31	0.30
316	Q9BTC0	DIDO1_HUMAN	Death-inducer obliterator 1	leakage														9431.31	11865.25	11528.70	9263.51	11223.17	11959.76	10878.62	0.11	16801.63	13264.23	14453.09	16526.89	12767.96	13654.85	14578.11	0.12	35776.31	63028.74	36736.14	44275.64	42097.30	58707.16	46770.21	0.24
317	Q96HQ0	ZN419_HUMAN	Zinc finger protein 419	leakage					x																									36426.19	50319.96	48458.02	36399.73	54317.54	39176.33	44182.96	0.18
318	P07996	TSP1_HUMAN	Thrombospondin-1	leakage					x																									44366.59	39432.54	21407.31	42368.52	47867.04	37342.60	38797.43	0.24
319	Q9BS31	ZN649_HUMAN	Zinc finger protein 649	leakage					x																									23893.29	41506.98	27029.68	51443.43	37055.76	43080.54	37334.95	0.28
320	A6NET4	OR5K3_HUMAN	Olfactory receptor 5K3	leakage					x																									38230.11	37448.37	43818.58	30572.98	25303.36	33517.48	34815.15	0.19
321	Q5VXU9	CI084_HUMAN	Uncharacterized protein C9orf84	not specified														2687.17	2791.06	4672.27	3239.96	3200.92	4463.52	3509.15	0.24									30866.18	33673.07	29242.40	27290.04	28598.75	24707.24	29062.94	0.11
322	Q8WXX0	DYH7_HUMAN	Dynein heavy chain 7, axonemal	leakage					x																									19153.12	26319.55	19664.16	22015.70	33036.85	19816.46	23334.31	0.23
323	O00507	USP9Y_HUMAN	Probable ubiquitin carboxyl-terminal hydrolase FAF-Y	leakage														25997.57	21566.03	14385.52	28745.89	13289.00	16526.06	20085.01	0.32									17684.62	20947.25	18574.38	30001.95	18006.58	18494.30	20618.18	0.23
324	O15068	MCF2L_HUMAN	Guanine nucleotide exchange factor DBS	leakage														1767.57	1386.69	1576.68	1296.65	1595.95	1051.12	1445.78	0.18	28831.42	18524.46	8851.50	9245.57	20799.40	16850.10	17183.74	0.44	21812.24	23484.69	16610.59	21711.41	12114.19	15060.55	18465.61	0.24
325	Q7Z443	PK1L3_HUMAN	Polycystic kidney disease protein 1-like 3	leakage					x																									12597.37	7541.63	19654.10	27878.66	14350.64	4420.43	14407.14	0.59
326	P98196	AT11A_HUMAN	Probable phospholipid-transporting ATPase IH	leakage					x																									15646.60	10306.86	12192.01	15633.99	14152.30	17414.16	14224.32	0.18
327	Q14789	GOGB1_HUMAN	Golgin subfamily B member 1	leakage					x																									12261.28	12966.09	13244.50	12153.63	14912.46	16265.88	13633.97	0.12
328	P42336	PK3CA_HUMAN	Phosphatidylinositol 4,5-bisphosphate 3-kinase catalytic subunit alpha isoform	not specified														1124.73	1204.97	1176.08	824.87	2321.75	1106.22	1293.10	0.40									11994.04	11442.01	11447.95	12246.20	15593.28	14681.10	12900.76	0.14
329	P0C7H9	U17L7_HUMAN	Inactive ubiquitin carboxyl-terminal hydrolase 17-like protein 7	leakage					x																									9658.89	13699.85	9527.35	12943.56	16998.28	9665.34	12082.21	0.25
330	A3KN83	SBNO1_HUMAN	Protein strawberry notch homolog 1	not specified					x																									10064.78	11306.97	12644.75	11854.82	10970.69	9867.64	11118.27	0.10
331	Q92623	TTC9A_HUMAN	Tetratricopeptide repeat protein 9A	not specified					x																									4561.18	14909.92	10024.42	13729.40	12358.96	9119.80	10783.95	0.35
332	P22105	TENX_HUMAN	Tenascin-X	secreted																						12453.55	10809.27	12486.04	14964.01	16866.78	13606.49	13531.02	0.16	11235.44	8385.27	10920.75	12733.07	8150.93	13159.06	10764.09	0.20
333	Q86T90	K1328_HUMAN	Protein hinderin	not specified														16908.72	16841.58	15622.29	15494.04	15313.31	13408.24	15598.03	0.08									9862.40	11562.00	10642.00	11316.39	12863.08	7463.62	10618.25	0.17
334	Q5TCS8	KAD9_HUMAN	Adenylate kinase 9	leakage					x																									9898.87	10199.37	11263.40	9480.61	10178.89	11514.74	10422.65	0.08
335	Q9UEF7	KLOT_HUMAN	Klotho	secreted																						17120.27	13483.25	17272.12	22997.85	21482.89	19907.31	18710.62	0.18	6675.10	9628.70	7123.88	17804.66	10324.22	9829.40	10230.99	0.39
336	O15273	TELT_HUMAN	Telethonin	leakage					x																									9032.56	11577.18	8591.26	8875.43	12078.15	7736.28	9648.48	0.18
337	Q9BSF8	BTBDA_HUMAN	BTB/POZ domain-containing protein 10	leakage					x																									9129.11	8307.40	7078.49	10871.11	7876.31	9212.97	8745.90	0.15
338	P01780	HV307_HUMAN	Immunoglobulin heavy variable 3-7	secreted																						371.99	290.86	428.77	469.32	322.08	557.09	406.69	0.24	7068.72	9527.41	5850.33	6734.04	8546.85	8393.02	7686.73	0.18
339	Q9Y2H2	SAC2_HUMAN	Phosphatidylinositide phosphatase SAC2	leakage					x																									5013.15	5941.13	6922.07	8951.12	6118.03	8374.53	6886.67	0.22
340	Q2M389	WASH7_HUMAN	WASH complex subunit SWIP	leakage														250.24	255.93	381.64	222.28	274.15	262.13	274.40	0.20									6085.25	6017.36	6883.56	6904.09	7154.24	7870.74	6819.21	0.10
341	Q9NQ86	TRI36_HUMAN	E3 ubiquitin-protein ligase TRIM36	leakage					x																									5319.77	6458.33	6188.87	7576.42	7692.69	7597.66	6805.62	0.14
342	P49454	CENPF_HUMAN	Centromere protein F	leakage					x																									6399.99	5736.58	6871.35	6709.91	5826.07	7427.01	6495.15	0.10
343	P57078	RIPK4_HUMAN	Receptor-interacting serine/threonine-protein kinase 4	leakage					x																									3484.21	5006.90	4886.33	8680.85	5964.93	7769.62	5965.47	0.33
344	O95568	MET18_HUMAN	Histidine protein methyltransferase 1 homolog	not specified					x																									4216.76	6174.97	5884.49	4732.16	7502.99	4704.26	5535.94	0.22
345	P02788	TRFL_HUMAN	Lactotransferrin	secreted					x																									3870.63	4644.48	4525.80	6649.32	5576.47	7176.62	5407.22	0.24
346	O43592	XPOT_HUMAN	Exportin-T	leakage					x																									2827.68	4834.92	2421.01	8204.64	4613.54	7644.30	5091.01	0.47
347	Q07864	DPOE1_HUMAN	DNA polymerase epsilon catalytic subunit A	leakage					x																									3913.72	4620.87	4648.20	4043.59	7816.98	4696.70	4956.68	0.29
348	O60282	KIF5C_HUMAN	Kinesin heavy chain isoform 5C	leakage														11134.27	9947.74	9590.92	11567.40	9997.11	15323.60	11260.17	0.19	313.79	355.80	355.26	402.09	633.23	417.34	412.92	0.28	3357.50	4705.72	4204.60	4241.52	4232.38	6382.07	4520.63	0.22
349	O94973	AP2A2_HUMAN	AP-2 complex subunit alpha-2	leakage					x																									4637.63	4978.92	5682.62	3686.25	2444.69	2844.34	4045.74	0.31
350	P38398	BRCA1_HUMAN	Breast cancer type 1 susceptibility protein	leakage																						0.01	15.98	88.66	180.67	19.29	248.19	92.13	1.10	3031.71	4225.44	3494.47	4681.33	4591.00	4132.33	4026.05	0.16
351	Q5W041	ARMC3_HUMAN	Armadillo repeat-containing protein 3	not specified					x																									1832.15	3018.80	1921.05	8672.56	3954.62	4733.67	4022.14	0.63
352	Q9P1Z3	HCN3_HUMAN	Potassium/sodium hyperpolarization-activated cyclic nucleotide-gated channel 3	leakage					x																									3240.91	4400.27	3598.47	4314.50	5135.56	3436.02	4020.95	0.18
353	A5A3E0	POTEF_HUMAN	POTE ankyrin domain family member F	leakage														513.40	757.78	686.85	600.90	733.27	611.00	650.53	0.14	0.01	103.43	115.44	261.28	112.43	262.63	142.54	0.72	5125.45	3622.05	3688.80	2590.60	4592.80	2445.16	3677.48	0.29
354	Q93008	USP9X_HUMAN	Probable ubiquitin carboxyl-terminal hydrolase FAF-X	leakage														353.70	383.22	430.03	388.72	281.15	456.80	382.27	0.16									2673.69	5102.35	4838.33	2527.33	3253.66	3080.53	3579.31	0.31
355	Q99973	TEP1_HUMAN	Telomerase protein component 1	leakage					x																									2872.29	2638.15	3056.81	3924.24	1561.96	2956.18	2834.94	0.27
356	O60308	CE104_HUMAN	Centrosomal protein of 104 kDa	leakage																						2449.36	3057.37	3402.56	3923.91	2862.61	3193.57	3148.23	0.16	2586.37	2072.08	4158.27	2740.11	2304.48	3072.79	2822.35	0.26
357	Q8NDV3	SMC1B_HUMAN	Structural maintenance of chromosomes protein 1B	leakage														892.99	1472.70	1320.55	1509.58	977.28	1594.79	1294.65	0.23	11245.13	12686.81	12878.75	13630.65	12753.01	12745.17	12656.59	0.06	2411.72	2593.27	2030.27	2757.18	2678.02	2978.64	2574.85	0.13
358	A6NMZ7	CO6A6_HUMAN	Collagen alpha-6	secreted														10517.35	12548.09	10190.11	13655.52	12731.93	10923.45	11761.07	0.12									2611.21	2538.41	2212.49	2273.77	2530.48	1975.30	2356.94	0.10
359	Q8ND61	CC020_HUMAN	Uncharacterized protein C3orf20	leakage														4917.49	3853.99	3776.39	3429.05	2906.28	2486.27	3561.58	0.24									1698.72	2021.20	1779.37	3094.01	2755.45	2400.67	2291.57	0.24
360	P48058	GRIA4_HUMAN	Glutamate receptor 4	leakage					x																									3077.93	1833.07	2699.30	2441.97	1328.52	2278.58	2276.56	0.27
361	Q13609	DNSL3_HUMAN	Deoxyribonuclease gamma	secreted					x																									1407.07	1437.72	1398.13	3589.14	2556.27	2736.01	2187.39	0.42
362	Q9BUN5	CC28B_HUMAN	Coiled-coil domain-containing protein 28B	leakage					x																									1642.63	2751.49	2034.47	2730.10	1852.01	2072.53	2180.54	0.21
363	P21359	NF1_HUMAN	Neurofibromin	leakage														11.11	12.77	50.14	5.12	11.88	46.96	23.00	0.87									1888.67	1314.30	2274.12	2784.22	1810.45	2908.72	2163.41	0.28
364	P02776	PLF4_HUMAN	Platelet factor 4	secreted														1913.44	4206.47	1289.93	2068.35	2625.21	1591.69	2282.51	0.46									2294.69	1964.33	2008.45	1930.65	2111.41	2394.68	2117.37	0.09
365	Q8NHQ9	DDX55_HUMAN	ATP-dependent RNA helicase DDX55	not specified					x																									1910.63	2073.88	2050.40	2381.40	1669.54	1970.65	2009.42	0.12
366	Q9NR63	CP26B_HUMAN	Cytochrome P450 26B1	leakage														366.79	189.64	307.12	369.23	163.70	371.54	294.67	0.32									1702.77	1800.04	1558.16	1903.05	2514.46	2106.02	1930.75	0.18
367	P04220	MUCB_HUMAN	Ig mu heavy chain disease protein	not specified																						746.88	399.32	864.08	997.54	1932.92	1344.03	1047.46	0.51	2045.65	1235.85	3234.22	1222.27	971.74	1488.23	1699.66	0.49
368	Q8IYG6	LRC56_HUMAN	Leucine-rich repeat-containing protein 56	not specified																						2317.57	2192.68	1620.26	2214.35	2071.65	4212.00	2438.08	0.37	1406.15	1656.27	1197.59	1361.52	1158.09	3402.11	1696.96	0.50
369	P40227	TCPZ_HUMAN	T-complex protein 1 subunit zeta	leakage					x																									1276.89	1346.32	1207.43	1743.68	2063.42	1974.73	1602.08	0.23
370	Q15166	PON3_HUMAN	Serum paraoxonase/lactonase 3	secreted														3111.16	4195.07	3826.46	6235.11	4290.51	5681.38	4556.62	0.26	816.16	792.14	2601.54	5369.21	4647.70	5314.42	3256.86	0.66	965.88	1217.40	1635.61	1651.54	1337.70	1217.99	1337.69	0.20
371	Q16706	MA2A1_HUMAN	Alpha-mannosidase 2	leakage					x																									1589.47	1410.83	1141.73	1127.97	1531.46	1125.96	1321.24	0.16
372	Q562R1	ACTBL_HUMAN	Beta-actin-like protein 2	leakage														380.27	439.00	986.80	745.44	691.00	982.28	704.13	0.37	1173.67	5514.73	8665.52	10939.20	8764.07	13023.34	8013.42	0.52	2042.17	539.77	1398.90	1463.94	1709.95	693.89	1308.10	0.45
373	P40197	GPV_HUMAN	Platelet glycoprotein V	leakage					x																									1157.34	1108.64	847.88	1767.30	1113.24	1464.71	1243.19	0.26
374	Q9Y4C1	KDM3A_HUMAN	Lysine-specific demethylase 3A	leakage														2592.48	2866.23	3260.40	4146.55	3325.19	3469.25	3276.68	0.16	2077.20	2363.66	1758.07	2012.62	1605.99	4069.94	2314.58	0.39	937.67	1169.85	1010.88	1375.56	1378.87	1494.22	1227.84	0.18
375	P63104	1433Z_HUMAN	14-3-3 protein zeta/delta	leakage					x																									834.30	1438.37	800.00	1065.62	1289.00	1654.25	1180.26	0.29
376	Q15485	FCN2_HUMAN	Ficolin-2	secreted					x																									1276.72	1108.32	1285.13	1120.29	839.13	1274.37	1150.66	0.15
377	Q9HAJ7	SP30L_HUMAN	Histone deacetylase complex subunit SAP30L	leakage					x																									698.16	973.13	888.53	1458.21	1258.23	1440.82	1119.51	0.28
378	Q12841	FSTL1_HUMAN	Follistatin-related protein 1	secreted					x																									895.59	783.04	1004.41	1174.44	1594.85	964.16	1069.41	0.27
379	P49908	SEPP1_HUMAN	Selenoprotein P	secreted					x																									1211.27	744.62	1239.40	927.55	693.97	922.78	956.60	0.24
380	P01591	IGJ_HUMAN	Immunoglobulin J chain	secreted					x																									879.22	1083.59	1054.26	825.27	705.08	654.64	867.01	0.20
381	Q5JVG2	ZN484_HUMAN	Zinc finger protein 484	leakage																						120.91	46.39	114.15	98.22	48.30	91.64	86.60	0.37	870.78	900.72	724.56	791.53	987.28	831.06	850.99	0.11
382	O60312	AT10A_HUMAN	Probable phospholipid-transporting ATPase VA	leakage					x																									465.01	533.37	569.41	1179.87	976.72	1181.35	817.62	0.41
383	P07359	GP1BA_HUMAN	Platelet glycoprotein Ib alpha chain	leakage					x																									750.91	861.62	617.19	990.96	805.81	751.14	796.27	0.16
384	H3BSY2	GOG8M_HUMAN	Golgin subfamily A member 8M	not specified																						5361.20	10026.18	7066.96	9134.62	8760.29	10406.44	8459.28	0.23	1023.79	658.78	531.43	935.06	444.49	1164.94	793.08	0.37
385	A4FU01	MTMRB_HUMAN	Myotubularin-related protein 11	not specified					x																									918.21	784.15	815.48	662.86	485.33	548.31	702.39	0.24
386	Q0VD83	APOBR_HUMAN	Apolipoprotein B receptor	leakage					x																									491.28	560.70	388.22	1162.87	638.84	825.75	677.94	0.41
387	Q8WTQ7	GRK7_HUMAN	Rhodopsin kinase	leakage					x																									634.57	539.33	784.97	650.49	615.25	746.00	661.77	0.14
388	Q8IYX3	CC116_HUMAN	Coiled-coil domain-containing protein 116	leakage					x																									590.82	609.21	358.38	689.62	923.17	621.96	632.19	0.29
389	Q9NPH3	IL1AP_HUMAN	Interleukin-1 receptor accessory protein	secreted					x																									462.11	416.45	1496.53	394.03	587.50	275.30	605.32	0.74
390	Q6UXB8	PI16_HUMAN	Peptidase inhibitor 16	secreted																						333.73	519.47	496.97	456.87	428.41	634.93	478.40	0.21	685.82	659.39	471.20	373.89	539.23	769.75	583.21	0.25
391	P61626	LYSC_HUMAN	Lysozyme C	secreted																						50.47	160.58	232.29	289.34	188.78	125.54	174.50	0.48	718.98	321.25	626.61	741.16	403.34	687.67	583.17	0.30
392	P11177	ODPB_HUMAN	Pyruvate dehydrogenase E1 component subunit beta, mitochondrial	leakage					x																									482.11	601.98	351.45	481.47	438.12	523.75	479.81	0.17
393	Q0D2K2	KLH30_HUMAN	Kelch-like protein 30	not specified					x																									565.84	396.41	618.41	439.35	407.96	287.32	452.55	0.27
394	P81605	DCD_HUMAN	Dermcidin	secreted					x																									291.37	161.41	861.62	286.68	664.39	404.32	444.97	0.60
395	Q9H4A6	GOLP3_HUMAN	Golgi phosphoprotein 3	leakage					x																									530.42	515.01	494.49	252.05	613.47	233.78	439.87	0.36
396	P42262	GRIA2_HUMAN	Glutamate receptor 2	leakage					x																									570.38	205.62	700.61	572.89	190.23	336.09	429.31	0.50
397	P62736	ACTA_HUMAN	Actin, aortic smooth muscle	leakage					x																									341.60	336.05	281.65	263.58	264.38	338.61	304.31	0.13
398	Q15020	SART3_HUMAN	Squamous cell carcinoma antigen recognized by T-cells 3	leakage					x																									445.71	180.57	268.68	301.44	119.56	229.15	257.52	0.44
399	P19012	K1C15_HUMAN	Keratin, type I cytoskeletal 15	not specified					x																									232.84	171.45	225.82	417.32	104.63	197.17	224.87	0.47

Supplementary material Overview on all sample collection methods relatively quantified proteins after depletion

Nr	Accession	Entry name	Protein names	secreted/ leakage	core set	unique EDTA	unique heparin	unique citrate	unique serum	EDTA plasma samples								heparin plasma samples								citrate plasma samples								serum samples							
										EDTA_1	EDTA_2	EDTA_3	EDTA_4	EDTA_5	EDTA_6	Mean abundance	CV	heparin_1	heparin_2	heparin_3	heparin_4	heparin_5	heparin_6	Mean abundance	CV	citrate_1	citrate_2	citrate_3	citrate_4	citrate_5	citrate_6	Mean abundance	CV	serum_1	serum_2	serum_3	serum_4	serum_5	serum_6	Mean abundance	CV
400	P01767	HV353_HUMAN	Immunoglobulin heavy variable 3-53	secreted					x																									400.71	19.44	368.65	311.11	170.96	50.89	220.29	0.74
401	P02100	HBE_HUMAN	Hemoglobin subunit epsilon	not specified																						238.98	434.56	490.83	720.59	523.69	739.65	524.72	0.36	159.32	238.13	222.18	190.26	169.85	184.00	193.96	0.16
402	Q03164	KMT2A_HUMAN	Histone-lysine N-methyltransferase 2A	leakage					x																									419.72	56.24	109.02	125.31	299.07	60.88	178.37	0.83
403	P54819	KAD2_HUMAN	Adenylate kinase 2, mitochondrial	leakage					x																									166.54	122.29	229.01	166.07	120.99	194.54	166.57	0.25
404	Q16236	NF2L2_HUMAN	Nuclear factor erythroid 2-related factor 2	leakage					x																									174.99	153.97	182.70	175.18	150.34	151.34	164.75	0.09
405	Q9ULC5	ACSL5_HUMAN	Long-chain-fatty-acid--CoA ligase 5	leakage					x																									91.75	44.55	84.41	88.24	201.43	105.95	102.72	0.51
406	Q96QR8	PURB_HUMAN	Transcriptional activator protein Pur-beta	leakage					x																									303.93	75.95	99.86	89.76	28.91	1.50	99.98	1.07
407	Q8WXW3	PIBF1_HUMAN	Progesterone-induced-blocking factor 1	secreted					x																									117.26	79.67	66.73	135.52	77.46	78.78	92.57	0.29
408	O43709	WBS22_HUMAN	Probable 18S rRNA	leakage					x																									84.23	43.69	93.93	108.65	61.60	102.49	82.43	0.30
409	Q9BXF6	RFIP5_HUMAN	Rab11 family-interacting protein 5	leakage					x																									34.90	12.18	24.62	63.94	19.76	40.91	32.72	0.56
410	Q86UW2	OSTB_HUMAN	Organic solute transporter subunit beta	leakage					x																									69.43	13.49	18.84	13.65	5.15	40.24	26.80	0.90
411	P48736	PK3CG_HUMAN	Phosphatidylinositol 4,5-bisphosphate 3-kinase catalytic subunit gamma isoform	leakage														65.05	96.97	101.40	127.34	50.97	328.91	128.44	0.79									37.32	8.69	7.97	11.49	8.33	3.54	12.89	0.95
412	Q9UHR6	ZNHI2_HUMAN	Zinc finger HIT domain-containing protein 2	not specified				x																		19442.65	16821.51	4967.25	3913.05	10581.18	11940.65	11277.72	0.55								
413	Q6ZS27	ZN662_HUMAN	Zinc finger protein 662	leakage				x																		938.45	1186.08	933.08	2114.85	653.04	2305.96	1355.24	0.51								
414	Q96RE9	ZN300_HUMAN	Zinc finger protein 300	leakage			x											7134.43	11365.11	11936.65	20051.35	15033.06	15472.04	13498.77	0.33																
415	Q8WU90	ZC3HF_HUMAN	Zinc finger CCCH domain-containing protein 15	leakage			x											2054.05	3192.74	2684.09	5334.58	2187.08	6026.44	3579.83	0.47																
416	A8MXQ7	YH010_HUMAN	Putative IQ motif and ankyrin repeat domain-containing protein LOC642574	not specified			x											2825.86	3244.49	2918.81	2643.17	2629.44	2655.71	2819.58	0.08																
417	Q8WTP9	XAGE3_HUMAN	X antigen family member 3	not specified				x																		76.70	260.70	672.89	779.32	554.45	483.20	471.21	0.56								
418	Q9H977	WDR54_HUMAN	WD repeat-containing protein 54	not specified				x																		1236.78	1715.98	1425.62	1373.79	1283.31	1602.31	1439.63	0.13								
419	P36543	VATE1_HUMAN	V-type proton ATPase subunit E 1	not specified				x																		3975.19	4535.47	5304.20	7910.39	3996.74	4033.76	4959.29	0.31								
420	Q6EMK4	VASN_HUMAN	Vasorin	secreted														910.84	954.98	811.52	620.76	650.24	1029.83	829.69	0.20	292.76	382.96	441.25	524.07	350.43	529.78	420.21	0.23								
421	Q9Y275	TN13B_HUMAN	Tumor necrosis factor ligand superfamily member 13B	secreted				x																		7559.92	5900.66	9551.76	8229.88	5886.75	5012.53	7023.58	0.24								
422	Q8N3R3	TCAIM_HUMAN	T-cell activation inhibitor, mitochondrial	leakage			x											13138.36	11100.95	8964.54	8622.09	9600.87	8056.75	9913.93	0.19																
423	Q71U36	TBA1A_HUMAN	Tubulin alpha-1A chain	leakage			x											2372.23	2808.85	2770.60	1537.66	2976.07	2458.90	2487.39	0.21																
424	Q8N4C7	STX19_HUMAN	Syntaxin-19	leakage				x																		28853.81	20450.99	15901.84	13099.16	22998.98	13805.55	19185.06	0.32								
425	Q13103	SPP24_HUMAN	Secreted phosphoprotein 24	secreted			x											365.54	302.09	68.21	114.56	227.63	75.83	192.31	0.65																
426	Q9HBM1	SPC25_HUMAN	Kinetochore protein Spc25	leakage				x																		143.26	95.06	3028.35	585.45	937.62	581.24	895.16	1.22								
427	Q8IW75	SPA12_HUMAN	Serpin A12	secreted			x											256.76	269.71	588.42	619.57	414.00	414.29	427.13	0.36																
428	O43166	SI1L1_HUMAN	Signal-induced proliferation-associated 1-like protein 1	leakage														9373.76	9043.80	9326.34	7494.31	7888.32	9501.03	8771.26	0.10	41253.87	23995.00	27240.11	27911.80	31691.21	32541.45	30772.24	0.20								
429	Q9P0W5	SCHI1_HUMAN	Schwannomin-interacting protein 1	leakage			x											17022.37	10273.26	10230.70	14051.27	10710.16	10656.20	12157.33	0.23																
430	P21673	SAT1_HUMAN	Diamine acetyltransferase 1	leakage			x											496.68	674.79	1381.39	474.87	497.13	1288.68	802.26	0.52																
431	P0DJI9	SAA2_HUMAN	Serum amyloid A-2 protein	secreted				x																		1362.57	1192.52	1434.70	2865.60	3447.40	3451.62	2292.40	0.47								
432	P25398	RS12_HUMAN	40S ribosomal protein S12	leakage			x											332.87	441.01	736.64	621.79	787.62	975.74	649.28	0.36																
433	Q9Y2L1	RRP44_HUMAN	Exosome complex exonuclease RRP44	leakage														4875.64	5867.98	8083.85	6393.71	9620.63	7779.54	7103.56	0.24	295.47	419.23	342.48	388.43	477.96	871.53	465.85	0.45								
434	Q7LG56	RIR2B_HUMAN	Ribonucleoside-diphosphate reductase subunit M2 B	leakage			x											5452.16	8108.55	8707.56	10249.58	8551.92	9234.59	8384.06	0.19																
435	Q5EBL4	RIPL1_HUMAN	RILP-like protein 1	leakage				x																		258.51	162.65	281.00	253.00	251.24	350.80	259.53	0.23								
436	Q9P2F6	RHG20_HUMAN	Rho GTPase-activating protein 20	not specified				x																		23588.91	22007.21	16026.98	12854.52	27895.57	20017.25	20398.41	0.26								
437	P49798	RGS4_HUMAN	Regulator of G-protein signaling 4	not specified			x											64.96	214.81	296.78	111.37	310.05	219.34	202.88	0.48																
438	Q8TAI7	REBL1_HUMAN	GTPase RhebL1	leakage				x																		5953.32	5546.38	4079.29	3589.31	4047.09	2788.38	4333.96	0.28								
439	Q15293	RCN1_HUMAN	Reticulocalbin-1	leakage				x																		3333.98	2570.87	2169.42	2829.25	9950.48	2626.29	3913.38	0.76								
440	Q5RL73	RBM48_HUMAN	RNA-binding protein 48	not specified			x											5453.95	8423.91	11530.65	14459.76	8906.23	21118.23	11648.79	0.48																
441	Q7Z444	RASE_HUMAN	GTPase ERas	leakage			x											27.35	100.35	633.34	314.32	249.07	569.50	315.65	0.78																
442	Q8WUY3	PRUN2_HUMAN	Protein prune homolog 2	leakage			x											160723.02	206288.30	192572.63	306294.22	247157.83	283486.75	232753.79	0.24																
443	Q6NUP7	PP4R4_HUMAN	Serine/threonine-protein phosphatase 4 regulatory subunit 4	leakage														11678.57	12490.37	11658.34	10382.64	10316.10	10136.79	11110.47	0.09	920.22	550.23	833.41	2267.39	1494.39	1139.08	1200.78	0.51								
444	P41247	PLPL4_HUMAN	Patatin-like phospholipase domain-containing protein 4	leakage			x											331.36	205.40	329.84	154.05	220.68	309.87	258.53	0.29																
445	Q8TBX8	PI42C_HUMAN	Phosphatidylinositol 5-phosphate 4-kinase type-2 gamma	leakage														107.10	130.96	182.34	69.66	203.29	148.10	140.24	0.35	56.27	138.79	218.47	134.41	156.31	103.34	134.60	0.40								
446	Q16816	PHKG1_HUMAN	Phosphorylase b kinase gamma catalytic chain, skeletal muscle/heart isoform	not specified			x											2272.53	2739.34	6269.82	2555.68	2796.17	5246.29	3646.64	0.46																
447	Q96RG2	PASK_HUMAN	PAS domain-containing serine/threonine-protein kinase	leakage				x																		6478.60	5954.96	8749.03	6905.58	9559.54	7206.75	7475.75	0.19								
448	O00750	P3C2B_HUMAN	Phosphatidylinositol 4-phosphate 3-kinase C2 domain-containing subunit beta	leakage			x											767.91	841.84	781.02	675.00	648.84	720.74	739.23	0.10																
449	Q99784	NOE1_HUMAN	Noelin	secreted			x											43.78	85.49	49.33	27.12	41.96	92.42	56.68	0.46																
450	Q9UKN7	MYO15_HUMAN	Unconventional myosin-XV	leakage			x											6640.25	7041.92	8641.70	6576.98	8135.98	6907.37	7324.03	0.12																
451	Q9H6N6	MYH16_HUMAN	Putative uncharacterized protein MYH16	not specified			x											2506.38	2784.98	2139.46	2955.12	3330.38	2449.90	2694.37	0.16																
452	Q9Y5Y7	LYVE1_HUMAN	Lymphatic vessel endothelial hyaluronic acid receptor 1	leakage				x																		916.60	521.19	463.60	680.42	1526.12	851.16	826.52	0.47								
453	P80748	LV321_HUMAN	Immunoglobulin lambda variable 3-21	secreted				x																		496.79	379.32	228.27	505.53	133.24	401.10	357.37	0.42								
454	Q99538	LGMN_HUMAN	Legumain	leakage				x																		1076.30	551.41	691.65	1184.16	719.05	581.34	800.65	0.33								
455	P24043	LAMA2_HUMAN	Laminin subunit alpha-2	secreted			x											11391.62	12287.63	14534.27	17687.75	16610.19	15376.19	14647.94	0.17																
456	O00515	LAD1_HUMAN	Ladinin-1	secreted			x											2808.30	3982.01	3469.03	2517.24	6132.14	2045.72	3492.41	0.42																
457	P01594	KV133_HUMAN	Immunoglobulin kappa variable 1-33	secreted			x											339.48	187.85	204.17	111.84	137.49	183.03	193.98	0.41																
458	P01602	KV105_HUMAN	Immunoglobulin kappa variable 1-5	secreted														63.95	120.64	528.50	196.01	122.95	505.01	256.18	0.81	460.07	351.56	2999.96	925.75	1913.67	1687.50	1389.75	0.73								
459	Q14525	KT33B_HUMAN	Keratin, type I cuticular Ha3-II	not specified														0.90	0.00	15.50	0.00	2.87	0.02	3.21	1.90	407.39	562.25	1988.99	575.91	891.64	641.18	844.56	0.69								
460	P78386	KRT85_HUMAN	Keratin, type II cuticular Hb5	not specified				x																		675.41	629.72	4749.99	942.74	1751.81	816.85	1594.42	1.00								
461	Q9NSB2	KRT84_HUMAN	Keratin, type II cuticular Hb4	not specified			x											747.51	794.21	1654.02	763.07	634.02	1269.34	977.03	0.41																
462	P78385	KRT83_HUMAN	Keratin, type II cuticular Hb3	not specified				x																		0.01	0.01	2636.86	32.33	63.14	112.81	474.19	2.24								
463	O76013	KRT36_HUMAN	Keratin, type I cuticular Ha6	not specified														826.60	986.36	1190.08	679.62	1002.33	1179.04	977.34	0.20	2565.62	646.13	2050.84	3057.47	3325.27	2681.41	2387.79	0.40								
464	Q92764	KRT35_HUMAN	Keratin, type I cuticular Ha5	not specified				x																		8825.94	16809.56	13368.93	18144.67	14259.95	15628.91	14506.33	0.23								
465	A6NCN2	KR87P_HUMAN	Putative keratin-87 protein	not specified				x																		0.01	9.78	1146.13	18.86	23.53	46.50	207.47	2.22								
466	Q5XKE5	K2C79_HUMAN	Keratin, type II cytoskeletal 79	not specified			x											31.72	34.88	71.68	31.35	1571.54	64.47	300.94	2.07																
467	Q86Y46	K2C73_HUMAN	Keratin, type II cytoskeletal 73	not specified			x											1008.94	1031.73	865.19	920.67	1190.03	1076.90	1015.58	0.11																
468	Q3SY84	K2C71_HUMAN	Keratin, type II cytoskeletal 71	leakage				x																		12.74	22.84	109.47	87.63	327.49	56.71	102.81	1.13								
469	P04259	K2C6B_HUMAN	Keratin, type II cytoskeletal 6B	not specified														996.42	1090.23	1195.85	1256.45	5718.19	1092.26	1891.57	0.99	864.02	722.42	640.91	790.51	745.60	1003.97	794.57	0.16								
470	P12035	K2C3_HUMAN	Keratin, type II cytoskeletal 3	not specified				x																		1686.28	1171.13	1318.85	1697.32	4501.18	2233.68	2101.41	0.59								
471	Q7Z3Y7	K1C28_HUMAN	Keratin, type I cytoskeletal 28	leakage			x											3820.95	5378.72	4191.35	3652.23	4050.23	4067.42	4193.48	0.15																
472	Q99456	K1C12_HUMAN	Keratin, type I cytoskeletal 12	not specified				x																		58.52	15.37	575.15	1016.67	741.02	813.00	536.62	0.77								
473	Q15811	ITSN1_HUMAN	Intersectin-1	leakage			x											50959.30	63100.20	53081.15	48219.67	90430.72	34338.54	56688.26	0.33																
474	Q9Y5U9	IR3IP_HUMAN	Immediate early response 3-interacting protein 1	leakage			x											2461.11	2005.70	1385.85	2761.53	1184.00	2471.32	2044.92	0.31																
475	P01877	IGHA2_HUMAN	Ig alpha-2 chain C region	not specified			x											467.07	662.74	577.10	532.16	477.39	557.76	545.70	0.13																
476	Q8WWN9	ICEF1_HUMAN	Interactor protein for cytohesin exchange factors 1	leakage			x											3660.44	2940.20	2614.38	4162.27	2188.94	3113.46	3113.28	0.23																
477	P83105	HTRA4_HUMAN	Serine protease HTRA4	secreted			x											61.45	57.17	50.69	50.30	42.03	59.21	53.48	0.13																
478	O60506	HNRPQ_HUMAN	Heterogeneous nuclear ribonucleoprotein Q	leakage			x											410.36	617.16	842.14	373.72	742.12	646.76	605.38	0.30																
479	P82970	HMGN5_HUMAN	High mobility group nucleosome-binding domain-containing protein 5	leakage				x																		9547.56	8133.30	7295.22	6810.45	7588.35	5103.05	7412.99	0.20								
480	P69891	HBG1_HUMAN	Hemoglobin subunit gamma-1	not specified														2707.93	3050.98	2516.51	1886.95	3195.58	2257.19	2602.52	0.19	1700.69	1689.45	2158.24	2565.07	3648.13	2716.44	2413.00	0.31								
481	Q58FF7	H90B3_HUMAN	Putative heat shock protein HSP 90-beta-3	leakage			x											7111.38	7132.19	6257.32	5701.62	6473.91	5482.06	6359.75	0.11																
482	P62805	H4_HUMAN	Histone H4	leakage				x																		10.71	16.68	346.46	2.13	3.49	2.52	63.66	2.18								
483	Q9UKJ3	GPTC8_HUMAN	G patch domain-containing protein 8	not specified				x																		1233.68	1382.57	1158.67	1798.83	1791.17	1491.99	1476.15	0.18								
484	A6NCC3	GOG8O_HUMAN	Golgin subfamily A member 8O	not specified														4225.79	3760.56	3202.72	2777.17	4164.38	1633.30	3293.99	0.30	749.00	483.74	583.47	758.78	1136.98	774.42	747.73	0.30								
485	Q99445	GML_HUMAN	Glycosyl-phosphatidylinositol-anchored molecule-like protein	leakage			x											230.50	336.32	383.63	275.61	346.93	311.86	314.14	0.17																
486	Q8WU03	GLYL2_HUMAN	Glycine N-acyltransferase-like protein 2	leakage														282.95	230.12	218.72	267.48	132.01	224.82	226.02	0.23	99.90	641.98	346.11	687.25	750.33	725.05	541.77	0.48								
487	P11413	G6PD_HUMAN	Glucose-6-phosphate 1-dehydrogenase	not specified			x											1111.48	896.98	1120.96	1170.24	1732.18	582.55	1102.40	0.34																
488	Q96IG2	FXL20_HUMAN	F-box/LRR-repeat protein 20	leakage			x											5.75	41.99	118.29	51.57	39.40	38.05	49.17	0.76																
489	Q8TBF8	FA81A_HUMAN	Protein FAM81A	not specified				x																		2397.41	14042.45	4878.81	6684.93	5054.50	6287.17	6557.55	0.60								
490	P12259	FA5_HUMAN	Coagulation factor V	secreted				x																		23814.19	17933.02	19660.08	25751.77	18389.29	25697.26	21874.27	0.17								
491	Q9UNN8	EPCR_HUMAN	Endothelial protein C receptor	leakage				x																		0.01	0.01	0.01	12.28	423.11	37.71	78.86	2.15								
492	Q96BY6	DOC10_HUMAN	Dedicator of cytokinesis protein 10	not specified				x																		3638.23	3355.05	4444.34	6537.16	8014.16	6194.95	5363.98	0.34								
493	P52429	DGKE_HUMAN	Diacylglycerol kinase epsilon	leakage			x											4955.07	4122.07	3520.68	3465.39	3319.32	3866.91	3874.91	0.16																

Supplementary material Overview on all sample collection methods relatively quantified proteins after depletion

Nr	Accession	Entry name	Protein names	secreted/ leakage	core set	unique EDTA	unique heparin	unique citrate	unique serum
494	Q9P219	DAPLE_HUMAN	Protein Daple	leakage			x		
495	P01036	CYTS_HUMAN	Cystatin-S	secreted				x	
496	O75638	CTAG2_HUMAN	Cancer/testis antigen 2	not specified					
497	Q9UPN4	CP131_HUMAN	Centrosomal protein of 131 kDa	leakage				x	
498	Q00610	CLH1_HUMAN	Clathrin heavy chain 1	leakage			x		
499	Q9P2M7	CING_HUMAN	Cingulin	leakage				x	
500	Q9UL16	CFA45_HUMAN	Cilia- and flagella-associated protein 45	leakage			x		
501	Q8IYT3	CC170_HUMAN	Coiled-coil domain-containing protein 170	not specified				x	
502	Q8NCX0	CC150_HUMAN	Coiled-coil domain-containing protein 150	not specified					
503	P49662	CASP4_HUMAN	Caspase-4	secreted			x		
504	Q96A19	C102A_HUMAN	Coiled-coil domain-containing protein 102A	not specified			x		
505	Q10588	BST1_HUMAN	ADP-ribosyl cyclase/cyclic ADP-ribose hydrolase 2	leakage			x		
506	Q9H3Q1	BORG4_HUMAN	Cdc42 effector protein 4	leakage				x	
507	P63267	ACTH_HUMAN	Actin, gamma-enteric smooth muscle	leakage					
508	Q9BYX7	ACTBM_HUMAN	Putative beta-actin-like protein 3	leakage				x	
509	Q15057	ACAP2_HUMAN	Arf-GAP with coiled-coil, ANK repeat and PH domain-containing protein 2	leakage					
510	P05534	1A24_HUMAN	HLA class I histocompatibility antigen, A-24 alpha chain	leakage			x		

Nr	EDTA plasma samples							
	EDTA_1	EDTA_2	EDTA_3	EDTA_4	EDTA_5	EDTA_6	Mean abundance	CV
494								
495								
496								
497								
498								
499								
500								
501								
502								
503								
504								
505								
506								
507								
508								
509								
510								

Nr	heparin plasma samples							
	heparin_1	heparin_2	heparin_3	heparin_4	heparin_5	heparin_6	Mean abundance	CV
494	7161.83	10199.62	14709.12	10856.80	11183.18	10820.41	10821.83	0.22
495								
496	165.09	42.77	140.12	110.61	86.56	188.22	122.23	0.44
497								
498	15585.80	14728.10	18561.65	20815.07	22935.44	20262.46	18814.75	0.17
499								
500	2848.39	4497.47	4272.40	3812.99	1646.42	4266.91	3557.43	0.31
501								
502	7443.48	8690.36	7846.42	6302.41	9490.72	7811.21	7930.77	0.14
503	776.07	704.78	837.21	1192.01	529.22	1844.42	980.62	0.49
504	1029.90	1121.45	838.28	620.37	856.36	893.37	893.29	0.19
505	1391.92	1511.85	1321.61	939.81	834.03	910.13	1151.56	0.25
506								
507	9637.57	8229.43	4836.29	7453.80	4734.93	5527.97	6736.67	0.30
508								
509	8354.38	10058.94	8393.02	8704.31	7595.70	9286.88	8732.20	0.10
510	413.09	460.73	483.77	360.86	343.48	406.01	411.33	0.13

Nr	citrate plasma samples							
	citrate_1	citrate_2	citrate_3	citrate_4	citrate_5	citrate_6	Mean abundance	CV
494								
495	11100.59	15658.52	13394.32	13585.52	12002.79	13466.96	13201.45	0.12
496	69015.53	46639.27	28351.79	21338.37	36097.45	20264.53	36951.15	0.50
497	253.79	556.04	571.39	623.02	433.58	787.55	537.56	0.34
498								
499	25421.93	20002.93	21886.01	23545.72	30961.39	24533.96	24391.99	0.15
500								
501	3005.07	2518.97	2760.20	2909.51	3691.98	2634.91	2920.11	0.14
502	5158.48	1596.71	6010.10	4326.18	4087.40	2576.83	3959.28	0.41
503								
504								
505								
506	0.01	0.01	21.92	58.20	27.51	40.08	24.62	0.93
507	11632.37	8455.75	8798.86	6868.74	10535.68	10119.66	9401.84	0.18
508	1814.76	2746.84	2981.03	3493.12	3782.09	3697.88	3085.95	0.24
509	61.45	24.03	309.96	490.26	486.00	184.08	259.29	0.79
510								

Nr	serum samples							
	serum_1	serum_2	serum_3	serum_4	serum_5	serum_6	Mean abundance	CV
494								
495								
496								
497								
498								
499								
500								
501								
502								
503								
504								
505								
506								
507								
508								
509								
510								
